# Supplementary material for: Structures of a non-ribosomal peptide synthetase condensation domain suggest the basis of substrate selectivity
Source: Nat Commun. 2021 May 4;12:2511. doi: 10.1038/s41467-021-22623-0 (PMC8097023; doi:10.1038/s41467-021-22623-0)
Supplement: Supplementary file 1 — Supplementary Information [file 41467_2021_22623_MOESM1_ESM.pdf]

Structures of a non-ribosomal peptide synthetase condensation domain suggest the basis of substrate selectivity

Izoré and Ho *et al.*

Supplementary information

Supplementary Tables

1. Data collection and refinement statistics (molecular replacement) Page 3
2. Similar structures to the PCP_2 domain (PDB ID 7KVW) as identified by DALI Page 4
3. Similar structures to the C_3 domain (PDB ID 7KVW) as identified by DALI Page 4
4. Interface residues in the PCP_2-C_3 complex as identified by PISA Page 5
5. Interactions between interface residues in the PCP_2-C_3 complex Page 5
6. Primer sequences and Spytag/Spycatcher sequences Page 6

Supplementary Discussion

Computational investigation of the mechanism of peptide bond formation Page 8

Supplementary Figures

1. Structural alignment (superposition) of the fuscachelin synthetase C_3 domain with C domains from surfactin and linear gramicidin Page 9
2. Superposition of PCP-C_3 structures that displays the PCP-orientation relative to the C domain Page 9
3. Superposition of the fuscachelin PCP2-C_3 didomain with C domains containing a PPant-modified PCP-domain (AB3403 and ObiF1) Page 10
4. Computational rigid body protein-protein docking of PCP_3 onto the acceptor PCP binding site of the C_3 domain Page 11
5. Tunnel analysis combined with molecular dynamics (MD) simulations highlight the intrinsic dynamics of the acceptor substrate tunnel Page 12
6. Sequence Logos of the R2577 residue of the PCP_2-C_3 didomain for C domains with ${}^{L}C_{L}$ selectivity, ${}^{D}C_{L}$ selectivity and starter C domains taken from the MiBiG database Page 13
7. Crystal structure of the R2577 mutant of the PCP_2-C_3 protein Page 14
8. Polder maps showing the electron density for the expected PCP-substrates Page 14
9. Chemical structures of Gly-CoA (aminoacyl-CoA) and the stabilized Gly_{stab}-CoA (modified Gly-CoA) Page 15
10. Interactions of R2577 with the PPant arm in the Gly_{stab} structure Page 15
11. Sequence Logos of the PPant interacting residues of the PCP_2-C_3 didomain for C domains with ${}^{L}C_{L}$ selectivity and ${}^{D}C_{L}$ selectivity taken from the MiBiG database Page 16
12. Comparison of the structures of a C domain containing a crosslinked acceptor mimic (PDB ID 5DU9) the Gly_{stab} structure (PDB ID 7KW0) Page 17
13. PPant ejection results showing extension of Gly_{stab} Page 18
14. Top poses of alternate acceptor substrates computationally docked into the C_3 domain Page 19
15. C domains do not appear have an "A domain like" side chain selection pocket for

their acceptor substrates Page 20
16. Stacked bar plots of substrate size percentage by mutation in the HHxxxDX motif Page 21
17. Glycine-PPant and Gly_{stab}-PPant computationally docked into WT and E2702G C_3 domains Page 22
18. Synthesis and characterization of BA-D-Arg-Gly-CoA Page 22
19. Synthesis and characterization of DHB-D-Arg-Gly-CoA Page 23
20. Synthesis and characterization of Gly_{stab}-CoA Page 23
21. Synthesis and characterization of d_4-Gly_{stab}-CoA Page 23
22. Synthesis and characterization of Gly-CoA Page 23
23. Synthesis and characterization of Ala-CoA Page 23
24. ^{1}H NMR spectrum of BA-D-Arg-Gly CoA Page 25
25. ^{1}H NMR spectrum of DHB-D-Arg-Gly CoA Page 26
26. ^{1}H NMR spectrum of Gly_{stab}-CoA Page 27
27. ^{1}H NMR spectrum of d_4-Gly_{stab}-CoA Page 28
28. ^{1}H NMR spectrum of Gly-CoA Page 29
29. ^{1}H NMR spectrum of Ala-CoA Page 30
30. LC-HRMS analyses for reconstitution of PCP2-C3::PCP3 WT without SpyCatcher and SpyTag Page 31
31. LC-HRMS analyses for reconstitution of PCP2-C3::PCP3 WT using SpyCatcher and SpyTag Page 32
32. LC-HRMS analyses for reconstitution of PCP2-C3::PCP3 WT using SpyCatcher and SpyTag Page 33
33. LC-HRMS analyses for reconstitution of PCP2-C3::PCP3 WT using SpyCatcher and SpyTag Page 35
34. LC-HRMS analyses for reconstitution of PCP2-C3::PCP3 WT using SpyCatcher and SpyTag Page 36
35. LC-HRMS analyses for reconstitution of PCP2-C3::PCP3 WT using SpyCatcher and SpyTag Page 37

Supplementary References Page 38

Supplementary Tables

Supplementary Table 1. Data collection and refinement statistics (molecular replacement)

	**WT PCP_2-C_3 PPant** (PDB 7KVW)	**WT PCP_2-C_3 Gly_{stab}** (PDB 7KW0)	**R2577G PCP_2-C_3 PPant** (PDB 7KW2)	**PCP_3** (PDB 7KW3)
**Data collection**				
Space group	$P2_12_12_1$	$P2_12_12_1$	$P2_12_12_1$	$P4_332$
Cell dimensions				
a, *b*, *c* (Å)	105.5, 105.9, 108.1	105.3, 105.5, 107.9	105.5, 106.1, 106.8	100.9, 100.9, 100.9
α, β, γ (°)	90, 90, 90	90, 90, 90	90, 90, 90	90, 90, 90
Resolution (Å)	48.14 - 2.18	48.04 - 1.90	47.68 - 2.00	45.13 - 2.30
R_{merge}	0.065 (0.72)	0.07 (1.46)	0.14 (1.6)	0.19 (2.5)
R_{pim}	0.032 (0.36)	0.032 (0.66)	0.06 (0.65)	0.03 (0.39)
$I / \sigma I$	12.4 (2.3)	13.3 (1.2)	5.8 (1)	24.3 (2.1)
$CC_{1/2}$	0.99 (0.71)	0.97 (0.50)	0.99 (0.52)	1 (0.73)
Completeness (%)	97.1 (93.4)	100 (100)	100 (99.9)	100 (99.7)
Redundancy	4.6 (4.6)	6.8 (6.9)	6.8 (6.8)	40.9 (41.6)
**Refinement**				
Resolution (Å)	47.4 - 2.18	48.0 – 1.90	47.7 – 2.0	45.1 – 2.3
No. reflections	61839	95147	81366	8248
R_{work} / R_{free}	0.19 / 0.23	0.19 / 0.22	0.19 / 0.22	0.18 / 0.2
No. atoms				
Protein	7952	7970	7917	573
Ppant/Gly_{stab}	42	48	42	-
Ion (SO_4)	-	-	-	5
Water	251	607	451	56
B-factors				
Protein	51.81	43.55	44.82	51.33
Ppant/Gly_{stab}	87.02	35.02	53.10	-
Ion (SO_4)	-	-	-	116.9
Water	50.69	44.29	48.18	58.2
R.m.s. deviations				
Bond lengths (Å)	0.003	0.004	0.005	0.006
Bond angles (°)	0.69	0.64	0.80	0.82

[a] Number of crystals = 1

Supplementary Table 2. Similar structures to the PCP_2 domain (PDB ID 7KVW) as identified by DALI.[1]

	PDB-ID	Z-score	RMSD	% identity	Description
1	6VTJ-A	14.4	1.3	32	PCP-R NON-RIBOSOMAL PEPTIDE SYNTHETASE[*]
2	4ZXI-A	13.8	1.2	31	HOLO-AB3403 C-A-PCP-TE[2]
3	4MRT-C	13.8	1.5	31	SFP/PCP COMPLEX[3]
4	4ZXH-A	13.7	1.3	31	HOLO-AB3403 C-A-PCP-TE[2]
5	4NEO-A	13.4	1.1	28	BLEOMYCIN NRPS TYPE II-PCP[4]
6	3TEJ-B	13.3	1.3	35	PCP/ TE COMPLEX [5]
7	2JGP-A	13.3	1.4	42	TYCC5-6 PCP-C NRPS DIDOMAIN[6]
8	5T3D-A	13.2	1.4	36	HOLO-ENTF NRPS[2]

*unpublished

Supplementary Table 3. Similar structures to the C_3 domain (PDB ID 7KVW) as identified by DALI.[1]

	PDB-ID	Z-score	RMSD	% identity	Description
1	4TX2-B	43.4	2.6	34	TEICOPLANIN X-DOMAIN[7]
2	6MFY-A	43.3	2.3	27	LINEAR GRAMICIDIN SYNTHASE SUBUNIT A[8]
3	6MFW-A	42.6	2.3	27	LINEAR GRAMICIDIN SYNTHASE SUBUNIT A[8]
4	4TX3-B	42.3	2.8	34	TEICOPLANIN X-DOMAIN / OXYB COMPLEX[7]
5	6MFX-A	41.9	2.3	27	LINEAR GRAMICIDIN SYNTHASE SUBUNIT A[8]
6	2JGP-A	41	3.1	28	TYROCIDINE SYNTHETASE 3[6]
7	6M7L-B	40.7	3.3	34	KISTAMICIN X-DOMAIN[9]
8	6MFZ-A	40.7	2.3	27	LINEAR GRAMICIDIN SYNTHASE SUBUNIT A[8]

Supplementary Table 4. Interface residues in the PCP_2-C_3 complex as identified by PISA (PDB ID 7KVW chain A).[10]

PCP residues*	ASA	BSA	Buried Fraction	Δ^iG#
F2508	72.20	57.3	0.8	0.54
A2509	87.46	18.39	0.2	0.23
G2511	55.01	3.96	0.1	0.05
G2512	6.50	3.30	0.5	-0.04
H2513	106.14	33.38	0.3	0.23
S2514	54.01	24.98	0.5	0.34
L2515	134.80	70.67	0.5	1.06
L2518	96.57	53.84	0.6	0.86
R2519	135.36	19.66	0.1	-0.08
V2534	29.82	22.68	0.8	0.36
R2535	180.72	83.47	0.5	0.40
F2538	113.79	92.67	0.8	1.23
E2539	136.65	40.45	0.3	-0.13
**C-domain residues***	**ASA**	**BSA**	**Buried Fraction**	**Δ^iG#**
R2576	100.93	3.22	0.1	-0.03
W2579	28.16	8.45	0.3	0.14
L2580	95.62	64.60	0.7	1.03
Q2583	98.71	19.38	0.2	0.11
V2584	87.07	53.57	0.6	0.84
D2630	158.67	20.61	0.2	-0.21
D2631	148.29	7.89	0.1	-0.09
G2632	17.44	11.88	0.6	0.19
P2633	19.32	12.22	0.7	0.20
A2783	89.79	19.70	0.2	0.17
T2784	97.52	24.26	0.2	0.39
D2900	72.84	33.29	0.5	-0.16
K2903	147.00	48.49	0.3	-0.22
R2906	138.20	108.61	0.8	-0.45
A2907	43.76	32.72	0.7	0.50
V2908	81.67	67.39	0.8	1.02
S2909	23.81	3.43	0.1	-0.04

* residues highlighted in green are central to the PCP/C hydrophobic interface

\# indicates the solvation energy of the corresponding residue, in kcal/M. The solvation energy gain of the interface is calculated as difference in solvation energies of all residues between dissociated and associated (interfacing) structures. Therefore, positive solvation energy Δ^iG of a residue makes a negative contribution to the solvation energy gain of the interface, which corresponds to hydrophobic effect. See reference #10.

Supplementary Table 5. Interactions between interface residues (identified by PISA)[10] in the PCP_2-C_3 complex (PDB ID 7KVW chain A).

PCP residue	C-domain residue	PCP residue	C-domain residue
F2508	R2906	L2518	W2579, L2580, Q2583, V2584
A2509	K2903	R2519	D2630, D2631
G2511	D2900	V2534	Q2583, V2584, V2908
G2512	D2900	R2535	A2783, T2784, V2908, S2909
H2513	D2900	F2538	V2584, R2906, A2907, V2908
S2514	V2584, R2906	E2539	A2783, V2908
L2515	R2576, W2579, L2580, D2631, G2632, P2633		

Supplementary Table 6. Primer sequences and Spytag/Spycatcher sequences used in this study.

Construct	Primer	Sequence
PCP_2-C_3 (V2481-A2803)	**1** (Fwd)	5’-GAACAGATCGGTGGTGTCACCGCCTACGAGGAGA-3’
	**2** (Rev)	5’-GTCTAGAAAGCTCTATGCCCCCGACACCACCT-3’
PCP_3 (pET28) (V3521-E3598)	**3** (Fwd)	5’-CACCATCACCATCACGGAAGCGTCCGCGAACCCGCAAC-3’
	**4** (Rev)	5’ GCCGGATCAAGCTTACTCGGTGACGGGCTGG-3’
PCP_3 (pHIS17) (V3521-E3598)	**5** (Fwd)	5’-GGATCCCATCATCATCATCATCATTAAAAGCT-3’
	**6** (Rev)	5’-ATGTATATCTCCTTCTTAAAGTTAAACAAAATTATTTCTAGAGGGA-3’
PCP_2-C_3 R2577G	**7** (Fwd)	5’-CACTTCCAGCGGGGGCTCTGGCTCA-3’
	**8** (Rev)	5’-TGAGCCAGAGCCCCCGCTGGAAGTG-3’
PCP_2-C_3 H2697Q	**9** (Fwd)	5’-TCGTCTGCCACCAGATTGCCGCAGACG-3’
	**10** (Rev)	5’-CGTCTGCGGCAATCTGGTGGCAGACGA-3’
PCP_2-C_3 E2702G	**11** (Fwd)	5’-CATTGCCGCAGACGGGTGGTCTTTCGCGC-3’
	**12** (Rev)	5’-GCGCGAAAGACCACCCGTCTGCGGCAATG-3’
pET28	**13** (Fwd)	5’-TAAGCTTGATCCGGCTGCTAACAA-3’
	**14** (Rev)	5’-GTGATGGTGATGGTGATGTTTCATGG-3’
pOPINS	**15** (Fwd)	5’-TAGAGCTTTCTAGACCATTTAAACACCACCAC
	**16** (Rev)	5’-ACCACCGATCTGTTCGCG
p17HIS	**17** (Fwd)	5’-GGATCCCATCATCATCATCATCATTAAAAGCT-3’
	**18** (Rev)	5’-ATGTATATCTCCTTCTTAAAGTTAAACAAAATTATTTCTAGAGGGA-3’
SpyCatcher	**19** (Fwd)	5’-GTGGTGTCGGGGGCAATGACAATTGAAGAAGATAGTGCTACCCA-3’
	**20** (Rev)	5’-GTCTAGAAAGCTCTAAATATGAGCGTCACCTTTAGTTGCTTTGC-3’
PCP_2C_3 in pOPINS - SpyCatcher	**21** (Fwd)	5’-TAGAGCTTTCTAGACCATTTAAACACCACCAC-3’
	**22** (Rev)	5’-TGCCCCCGACACCACCTC-3’
SpyTag	**23** (Fwd)	5’-GGAGATATACATATGGGAGCCCACATCGTG-3’
	**24** (Rev)	5’-TGCGGGTTCGCGGACACCACTTTCACCACTACCCTT-3’
PCP_3 in pHIS17 - SpyTag	**25** (Fwd)	5’-GTCCGCGAACCCGCAACC-3’
	**26** (Rev)	5’-CATATGTATATCTCCTTCTTAAAGTTAAACAAAATTATTTCTAGAGGGA-3’
Spycatcher	ATGACAATTGAAGAAGATAGTGCTACCCATATTAAATTCTCAAAACGTGATGAGGACGGCAAAGAGTTAGCTGGTGCAACTATGGAGTTGCGTGATTCATCTGGTAAAACTATTAGTACATGGATTTCAGATGGACAAGTGAAAGATTTCTACCTGTATCCAGGAAAATATACATTTGTCGAAACCGCAGCACCAGACGGTTATGAGGTAGCAACTGCTATTACCTTTACAGTTAATGAGCAAGGTCAGGTTACTGTAAATGGCAAAGCAACTAAAGGTGACGCTCATATT	
Spytag	GGAGCCCACATCGTGATGGTGGACGCCTACAAGCCGACGAAGGGTAGTGGTGAAAGTGGT	

Supplementary Discussion

Computational investigation of the mechanism of peptide bond formation. Density functional theory (DFT) computations were performed to explore the mechanism of peptide bond formation catalyzed by the C-domain. Two plausible mechanisms were studied. The first mechanism, shown in **Figure 5c** (upper pathway), was a concerted process in which the nucleophilic amine group is deprotonated by the active-site histidine residue at the same time as the amine attacks the thioester to form the new N–C bond. Our DFT calculations[11] with the B3LYP-D3 functional[12-16] and 6-31G(d) basis set in implicit diethyl ether (ε = 4.24, chosen to model the dielectric constant of the interior of an enzyme, and modelled with the SMD implicit model[17]) were unable to locate the transition state (TS) for the concerted process. Even in the presence of a small number of explicit water molecules to stabilize the developing oxyanion, these structures could not be located on the potential energy surface; they instead spontaneously collapsed to the transition states or intermediates of the alternative non-concerted mechanism. The second plausible mechanism, shown in **Figure 5c** (lower pathway), was a stepwise process. In this mechanism, the nucleophilic attack on the thioester and the deprotonation of the amine take place sequentially in separate chemical steps. In contrast to the concerted process, it was readily possible to calculate transition states and zwitterionic tetrahedral intermediates for this type of mechanism. We calculated several variants, differing with respect to the number and placement of water molecules around the oxyanion. With no water molecules located near the oxyanion, the zwitterionic intermediate was not stable and instead spontaneously dissociated back to the reactants. However, when one or more water molecules were present forming hydrogen bonds to the O^-, the zwitterion could be located as a stable structure. A variety of zwitterionic intermediates, and corresponding transition states, stabilized by one, two, or three water molecules, were located. A representative example is shown in **Figure 5c** (right hand side). Importantly, as illustrated by the structure in **Figure 5c**, even when the histidine was positioned close to the ammonium group of the zwitterion, proton transfer did not occur spontaneously. Instead, it took place in a separate chemical step with its own energy barrier. Based on these results, the most likely mechanism of peptide bond formation appears to be the sequential process shown in the lower pathway of **Figure 5c**. Whilst we have used water molecules as model hydrogen bond donors in our calculations, it is likely that in the enzyme active site, other hydrogen bond donors could alternatively serve in a similar role to stabilize the oxyanion. Furthermore, computations indicated that after the zwitterionic intermediate has been formed, the subsequent N-deprotonation and C–S cleavage processes could occur either in concert or sequentially.

Supplementary Figures

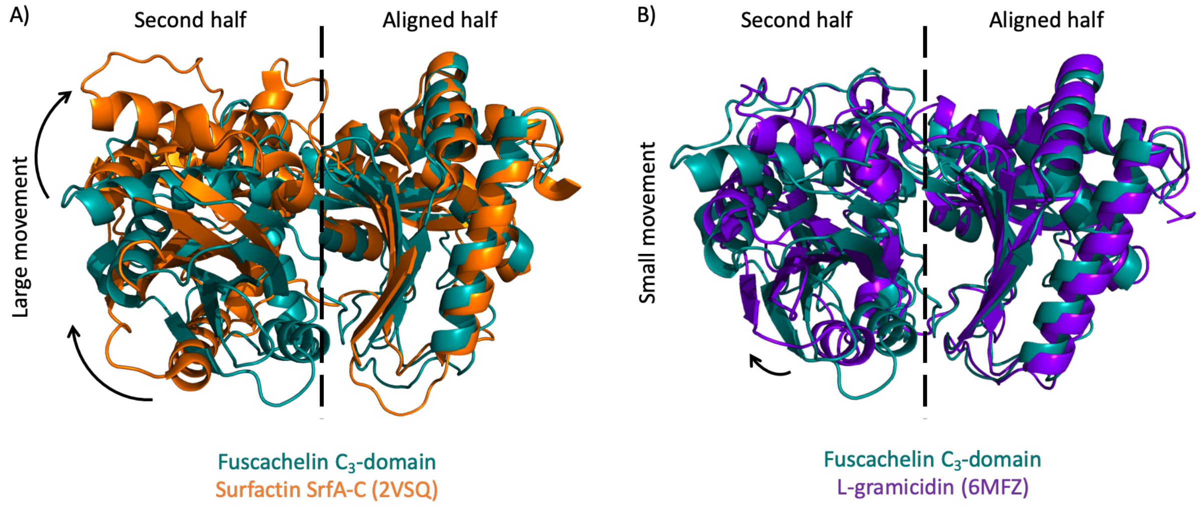

Supplementary Figure 1. Structural alignment (superposition) of the fuscachelin synthetase C_3 domain with C domains from surfactin and linear gramicidin. A) Structural alignment of the C-terminal half of fuscachelin synthetase C_3 domain (PDB ID 7KVW) with surfactin SrfA-C (2VSQ) showing a large movement of the N-terminal portion of the domain. B) Superposition of the C-terminal half of fuscachelin synthetase C_3 domain with L-gramicidin synthetase C-domain (6MFZ) showing a small movement of the N-terminal portion of the domain.

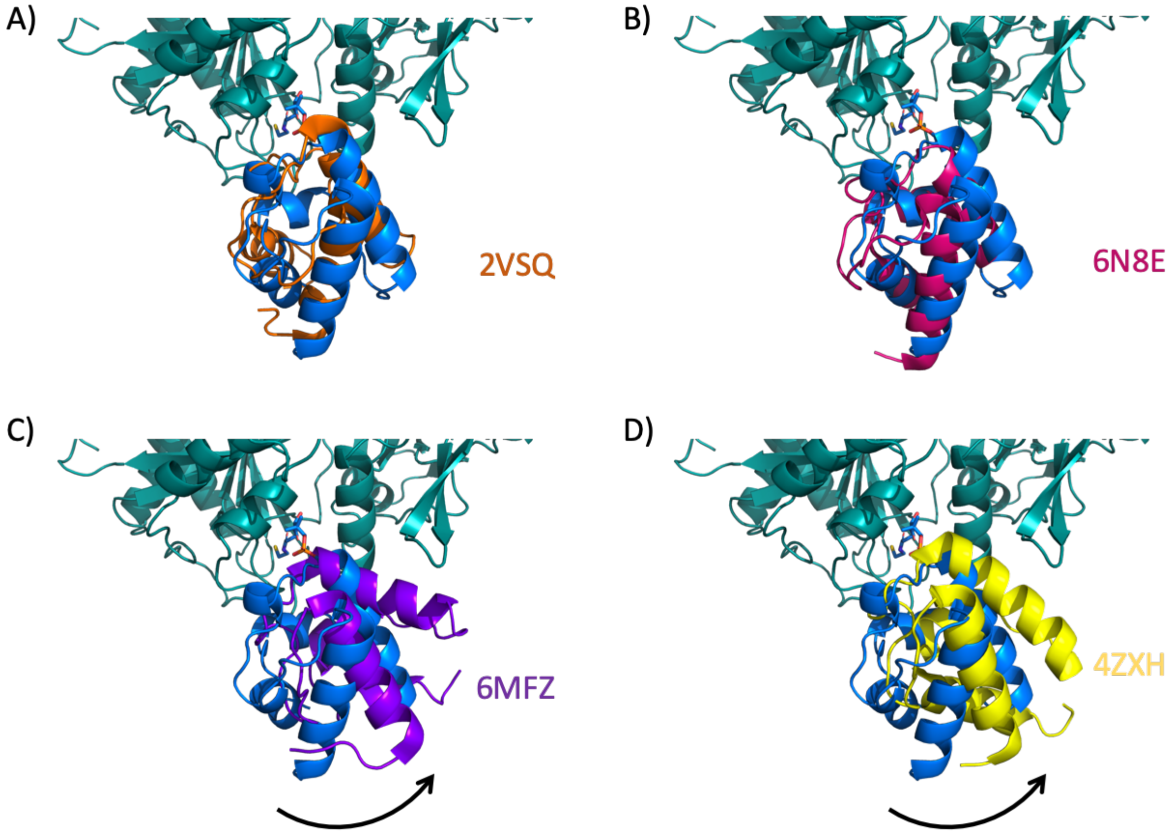

Supplementary Figure 2. Superposition of PCP-C structures that displays the PCP-orientation relative to the C domain. A) Superposition of the fuscachelin PCP_2-C_3 structure (PDB ID 7KVW) with SrfA-C (PDB ID 2VSQ). B) Superposition of the fuscachelin PCP_2-C_3 structure with ObiF1 (PDB ID 6N8E). C) Superposition of the fuscachelin PCP_2-C_3 structure with LgrA (PDB ID 6MFZ). D) Superposition of the fuscachelin PCP_2-C_3 structure with AB3403 (PDB ID 4ZXH). The structures of SrfA-C and ObiF1 are similar to the fuscachelin PCP_2 domain whereas AB3403 and LgrA display a different rotation around the conserved serine.

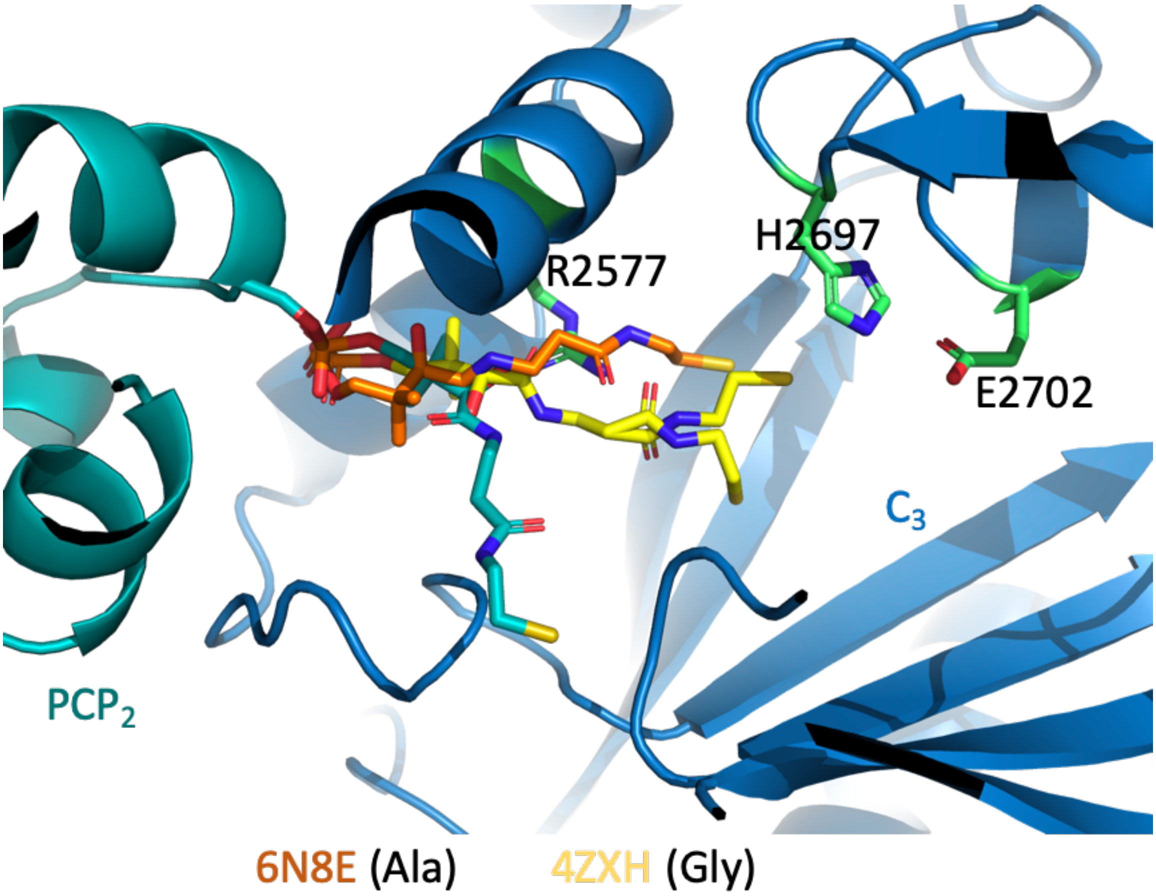

Supplementary Figure 3. Superposition of the fuscachelin PCP_2-C_3 didomain (PDB ID 7KVW) with C domains containing a PPant-modified PCP-domain (AB3403 (PDB ID 4ZXH) and ObiF1 (PDB ID 6N8E)). When comparing the structures, it is apparent that the R2577 side chain prevents PPant access to the C domain active site; in the other structures this is present as a small residue (see bottom of figure).

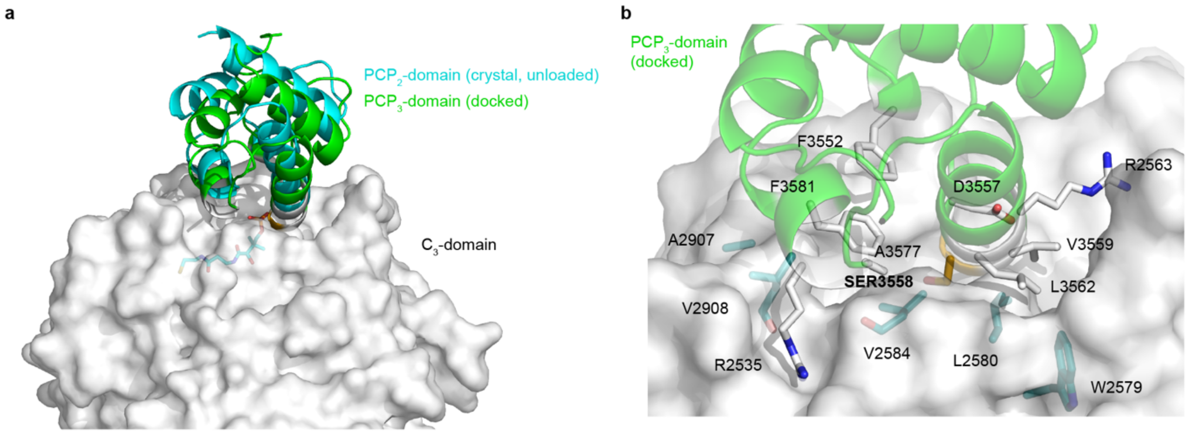

Supplementary Figure 4. Computational rigid body protein-protein docking of PCP_3 onto the acceptor PCP binding site of the C_3 domain. (a) Structural overlay of the top scoring pose of PCP_3 (7KW3) docked onto the acceptor PCP binding site of C_3-domain (green; from 7KVW) and the (unloaded) PCP_2-C_3 didomain complex (PCP_2 in cyan; 7KVW) as determined by computational rigid-body docking. (b) Residues at the interface between the computationally docked PCP_3 (top pose) and C_3. C_3 domain residues are colored teal, while key PCP_3 domain interface residues are shown as white sticks. The key serine residue (Ser3558) is colored orange.

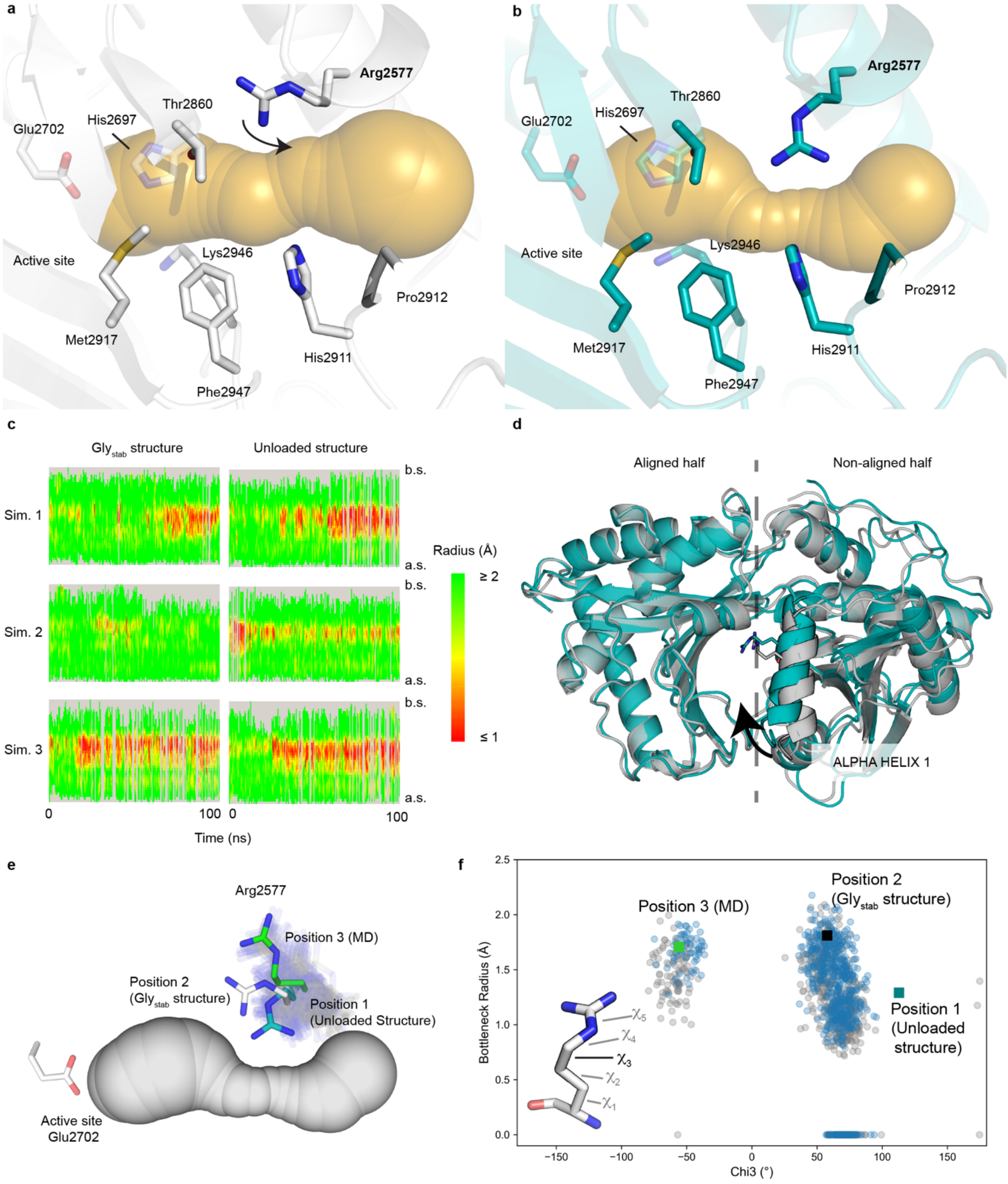

Supplementary Figure 5. Tunnel analysis combined with molecular dynamics (MD) simulations highlight the intrinsic dynamics of the acceptor substrate tunnel. (a-b) CAVER[18] tunnel analysis of the acceptor substrate tunnel of C_3 from the (a) Gly$_{stab}$-PPant crystal structure (PDB ID 7KW0 Chain A) and (b) unloaded PPant crystal structure (PDB ID 7KVW Chain A) highlight differences in the tunnel profiles (gold volumes) and that Arg2577 is a key bottleneck-lining residue. Simulations were initiated from the isolated structures of the C_3 domain, with the PCP domain and PPant moiety removed. (c) Heat-maps showing how the profile of the acceptor tunnel changes over the course of triplicate (n=3) 100 ns MD simulations initiated from these two structures (with PPants removed). The tunnel bottleneck (red) occurs halfway along the tunnel between the active site (a.s.) and bulk solvent (b.s.). (d)

Structural alignment of C_3 from the unloaded-PPant structure (Chain A, teal) and a snapshot taken from a MD simulation (grey) of this protein. The size of the acceptor tunnel is primarily determined by the rotameric state of Arg2577 (sticks), but is also influenced by larger scale motions, including the displacement of alpha helix 1. (e) During simulations, Arg2577 samples rotamers corresponding to those found in the unloaded PPant crystal structure (position 1, teal) and Gly_{stab} crystal structure (position 2, white), but also samples a number of other states (grey cloud), including a distinct third position (green, Chi3= -80° to -40°). (f) Analysis of snapshots from MD simulations of C_3 from the unloaded-PPant (grey) and Gly_{stab}-PPant (blue) structures show that when Arg2577 is in this third position the tunnel remains open (bottleneck radius > 1 Å). In contrast, rotation of Arg2577 towards the tunnel (Chi3 = 30° to 100°) is associated with smaller tunnels. Tunnels with bottleneck radii smaller than that of the probe sphere (0.7 Å) were given a bottleneck radius of 0 Å. Source Data for (c) and (f) available in the Source Data file.

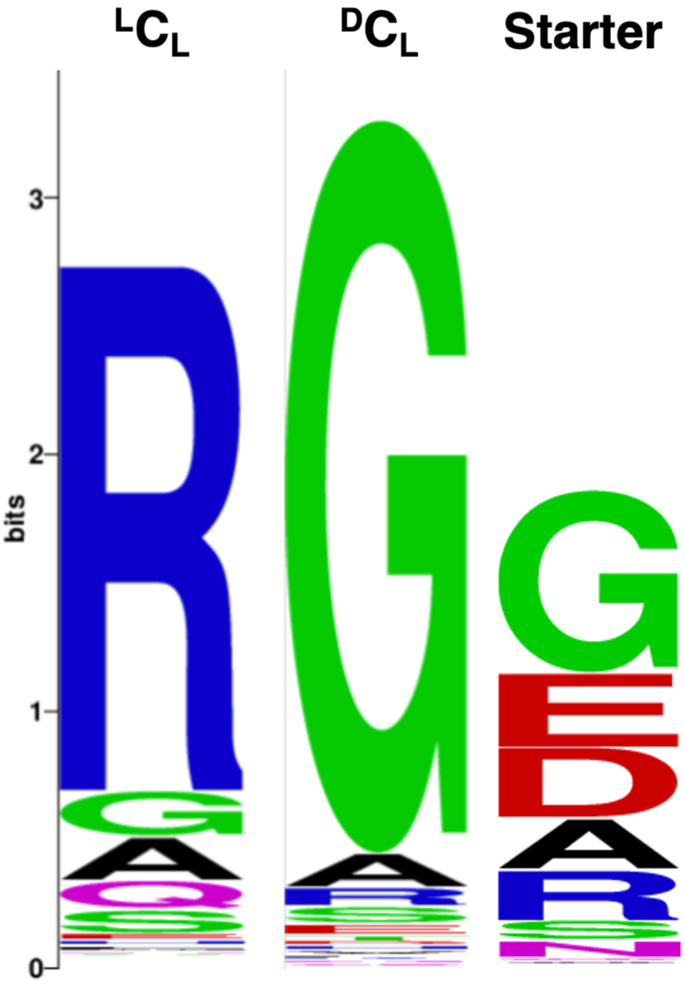

Supplementary Figure 6. Sequence Logos of the R2577 residue of the PCP_2-C_3 didomain for C domains with $^{L}C_{L}$ selectivity (1456 sequences), $^{D}C_{L}$ selectivity (593 sequences) and starter C domains (152 sequences) taken from the MiBiG database. The arginine residue is largely conserved (in 72.9% of cases found) in C domains conforming to $^{L}C_{L}$ selectivity, while glycine is the most prominent residue in cases of $^{D}C_{L}$ selectivity (in 80.1% of cases found).

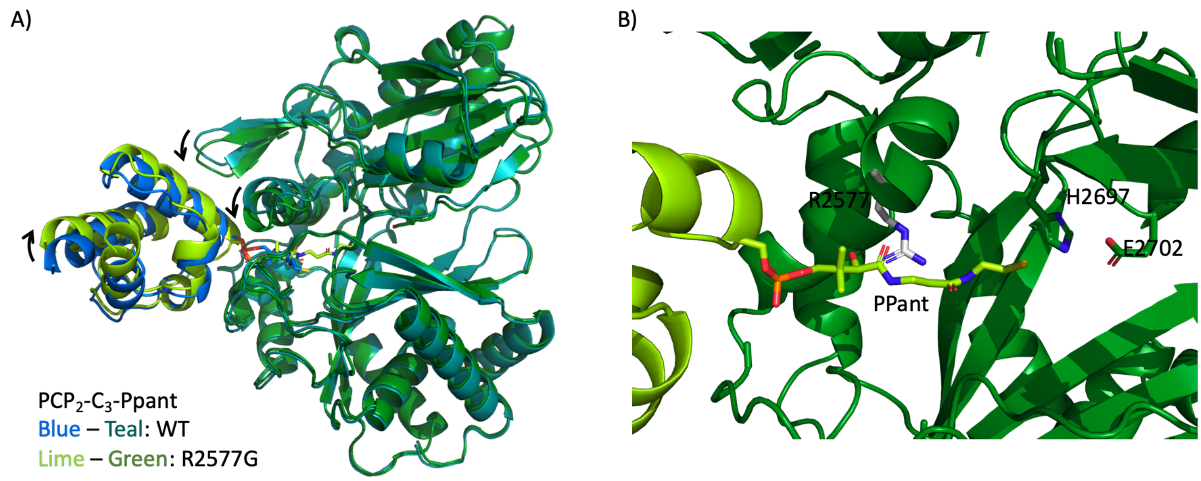

Supplementary Figure 7. Crystal structure of the R2577 mutant of the PCP_2-C_3 protein. A) Superposition of the R2577G mutant (PDB ID 7KW2) onto the wild type protein. B) Close-up of the acceptor channel with the PPant extending towards the catalytic site. The mutated arginine residue is shown as white sticks.

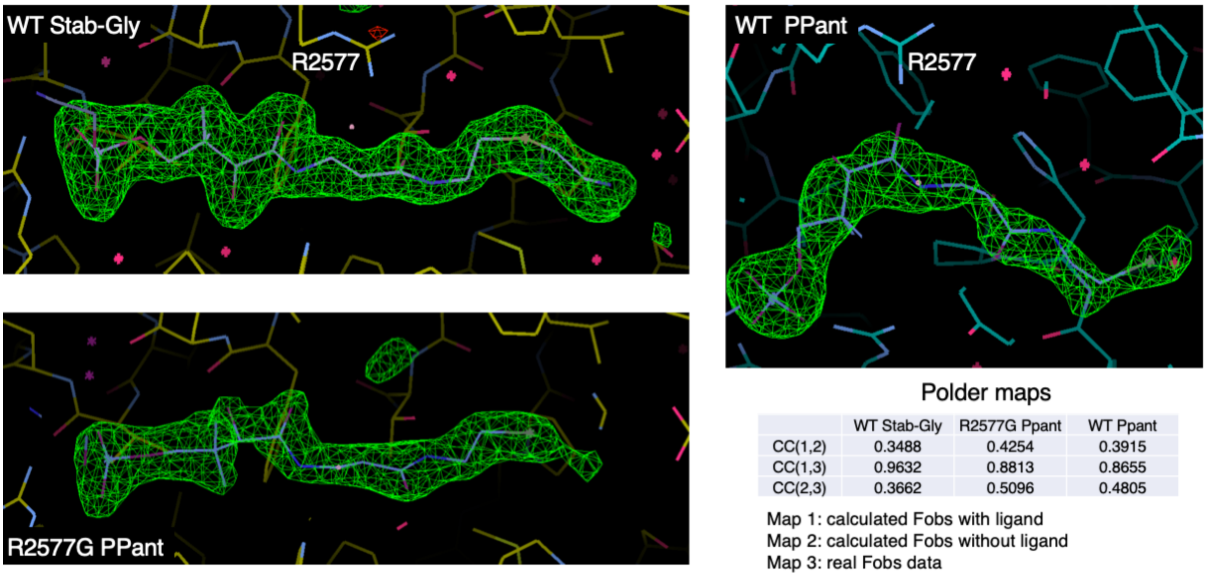

	WT Stab-Gly	R2577G Ppant	WT Ppant
CC(1,2)	0.3488	0.4254	0.3915
CC(1,3)	0.9632	0.8813	0.8655
CC(2,3)	0.3662	0.5096	0.4805

Map 1: calculated Fobs with ligand
Map 2: calculated Fobs without ligand
Map 3: real Fobs data

Supplementary Figure 8. Polder maps showing the electron density for the expected PCP-substrates. Correlation coefficients (CC) shown bottom right. WT Stab-Gly 7KW0, WT PPant 7KVW, R2577G PPant 7KW2.

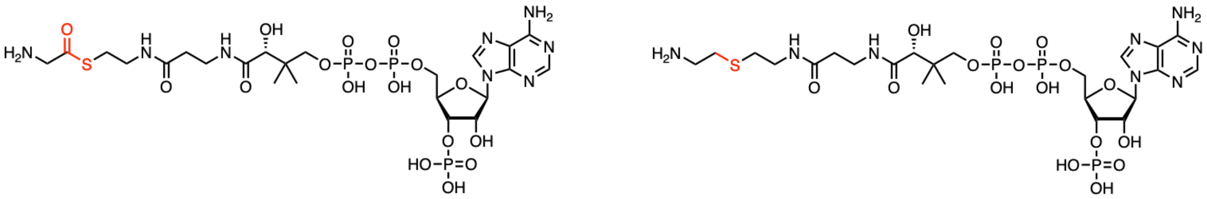

Gly-CoA   Gly$_{stab}$-CoA

Supplementary Figure 9. Chemical structures of Gly-CoA (aminoacyl-CoA) and the stabilized Gly$_{stab}$-CoA (modified Gly-CoA).

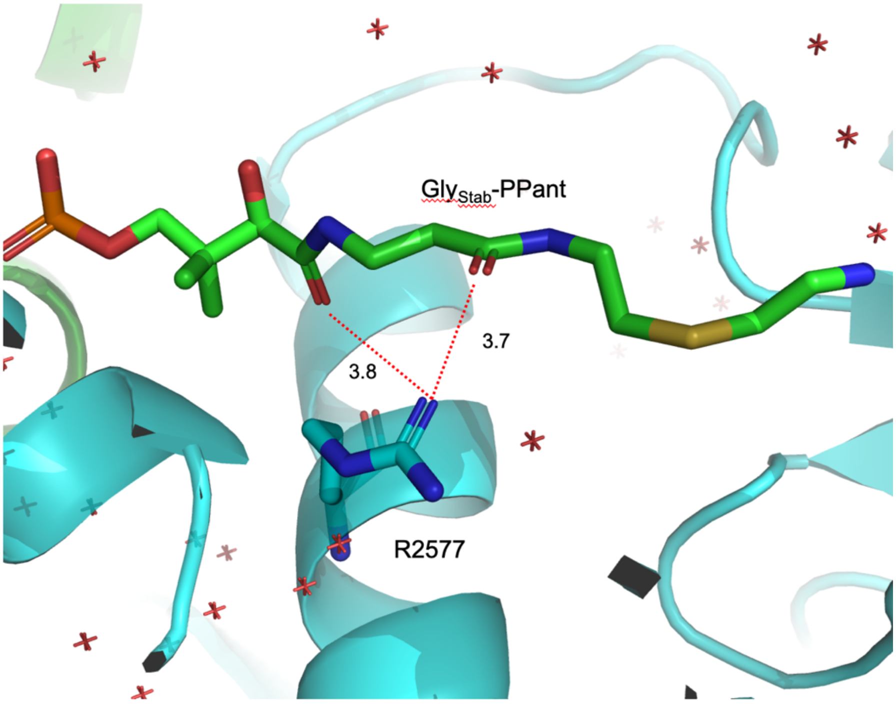

Supplementary Figure 10. Interactions of R2577 with the PPant arm in the Gly$_{stab}$ structure (7KW0). H-bonds shown as red dotted lines, distances indicated in Angstroms (Å).

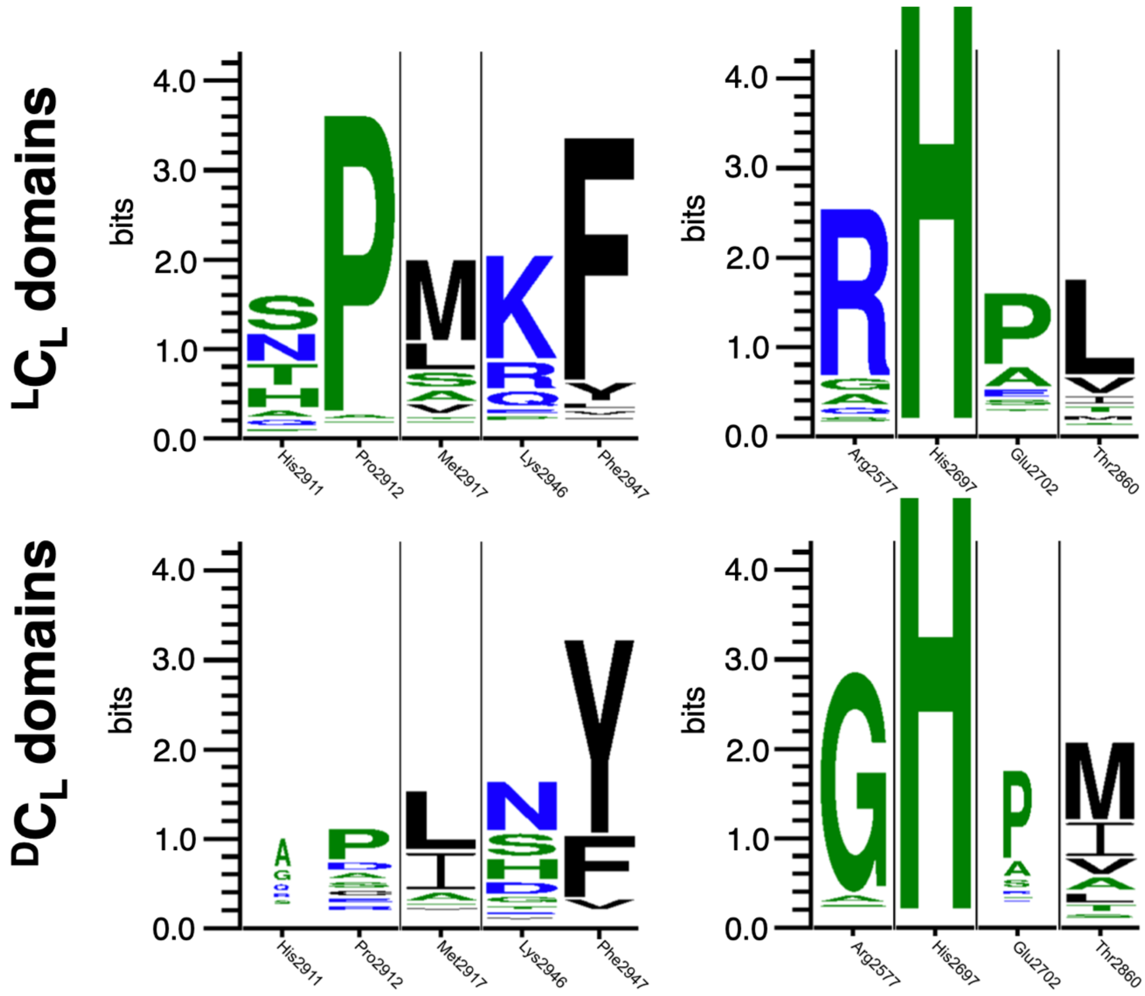

Supplementary Figure 11. Sequence Logos of the PPant interacting residues of the PCP_2-C_3 didomain for C domains with $^{L}C_{L}$ selectivity (350 sequences) and $^{D}C_{L}$ selectivity (113 sequences) taken from the MiBiG database. Residue numbering and identities taken from the fuscachelin PCP_2-C_3 sequence and structures.

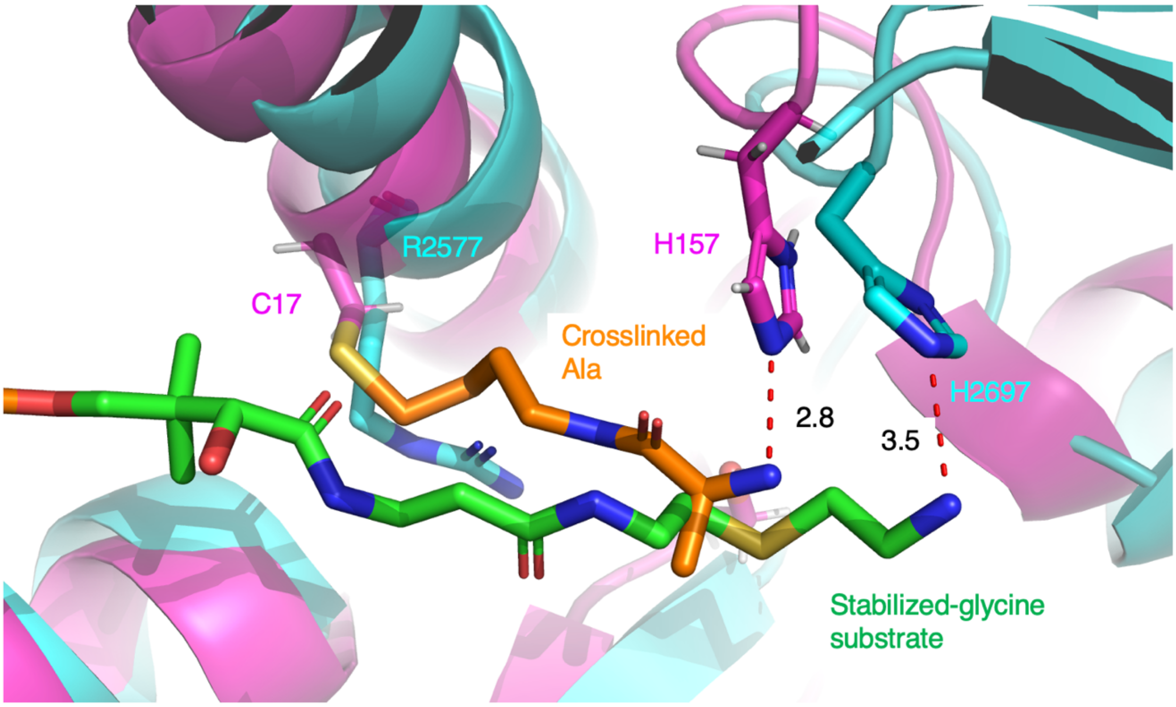

Supplementary Figure 12. Comparison of the structures of a C domain containing a crosslinked acceptor mimic (PDB ID 5DU9) the Gly$_{stab}$ structure (PDB ID 7KW0). The amine of the Gly$_{stab}$ structure enters the C domain active site by a further 3.6 Å than the crosslinked Ala moiety. 5DU9 structure shown in magenta with the acceptor mimic shown in orange sticks; 7KW0 structure shown in cyan with the PPant/Gly$_{stab}$ moiety shown in green sticks. H-bonds shown as red dotted lines, distances indicated in Angstroms (Å).

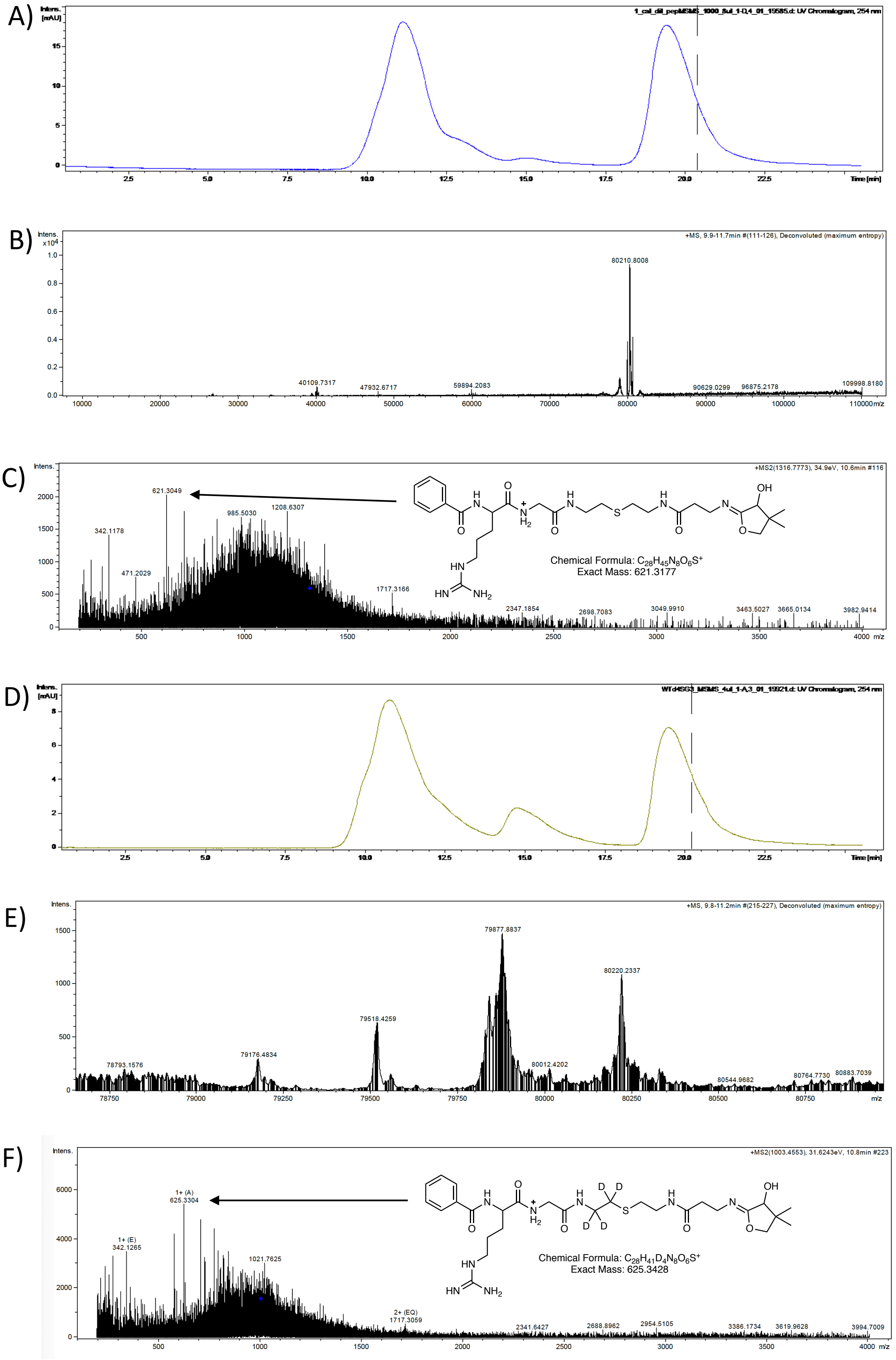

Supplementary Figure 13. PPant ejection results showing extension of Gly_{stab}. NanoLC-MS analyses indicating the reconstitution of PCP_2-C_3::PCP_3 WT for BA-D-Arg-Gly donor peptide together with

Gly_{stab}-CoA (A-C) or d_4-Gly_{stab}-CoA (D-F) as an acceptor substrate. A) UV trace of chromatogram with the protein eluting at 11 minutes. B) Deconvoluted spectrum showing the [M]+ masses observed. C) MS^2 spectrum of the 76+ charged ion 1056 (calculated for $C_{28}H_{45}N_8O_6S^+$ $[M+H]^+$: 621.32, found: 621.31). D) UV trace of chromatogram with the protein eluting at 11 minutes. E) Deconvoluted spectrum showing the [M]+ masses observed. F) MS^2 spectrum of the 80+ charged ion 1003 (calculated for $C_{28}H_{41}D_4N_8O_6S^+$ $[M+H]^+$: 625.34, found: 625.33). Use of the d_4-labeled substrate allowed confirmation of the identity of the PPant ejection peak due to the 4 Da shift in mass.

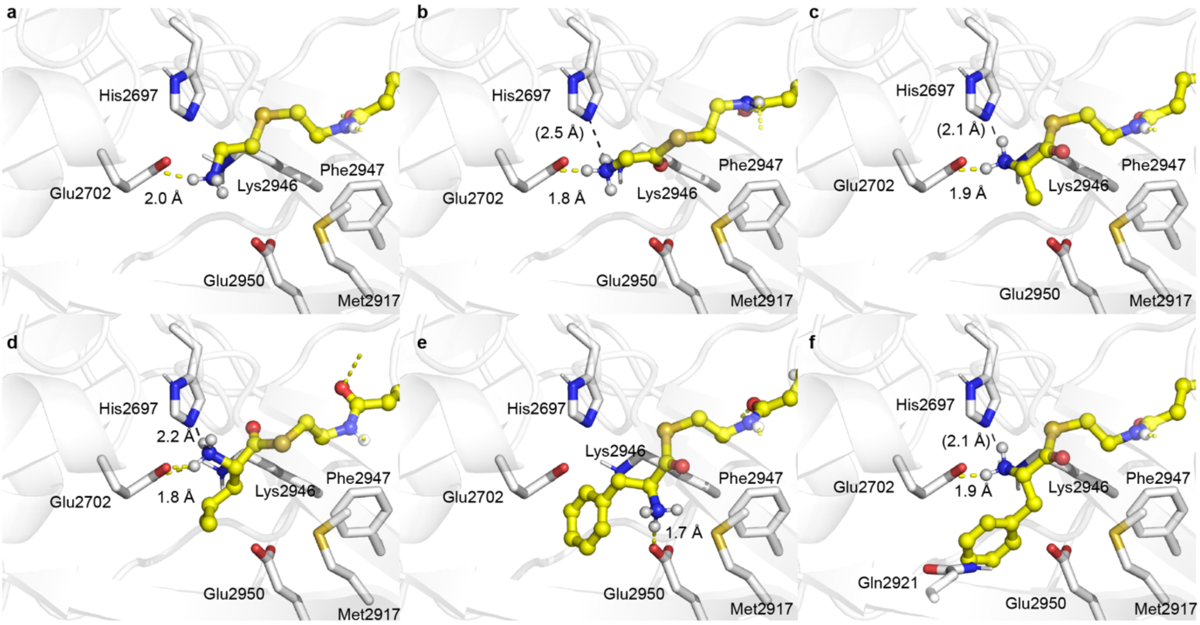

Supplementary Figure 14. Top poses of alternate acceptor substrates computationally docked into the C_3 domain (7KW2). Top-scoring poses of docked substrates in the C_3 domain (using chain A, residues 2558 – 2999 of the Gly_{stab} PCP_2-C_3 didomain structure). Panels show (a) Gly_{stab}, (b) Gly, (c) L-Ala, (d) L-Leu and (e) L-Phe docked into the C_3 domain. (f) Using the top pose of docked L-Ala as the template and manually building out the side chain of L-Phe, followed by testing of all possible rotamers showed that this bulky sidechain clashed with active site pocket residues when the amino acid backbone is restrained in the catalytically-competent position. Hydrogen bond distances indicated.

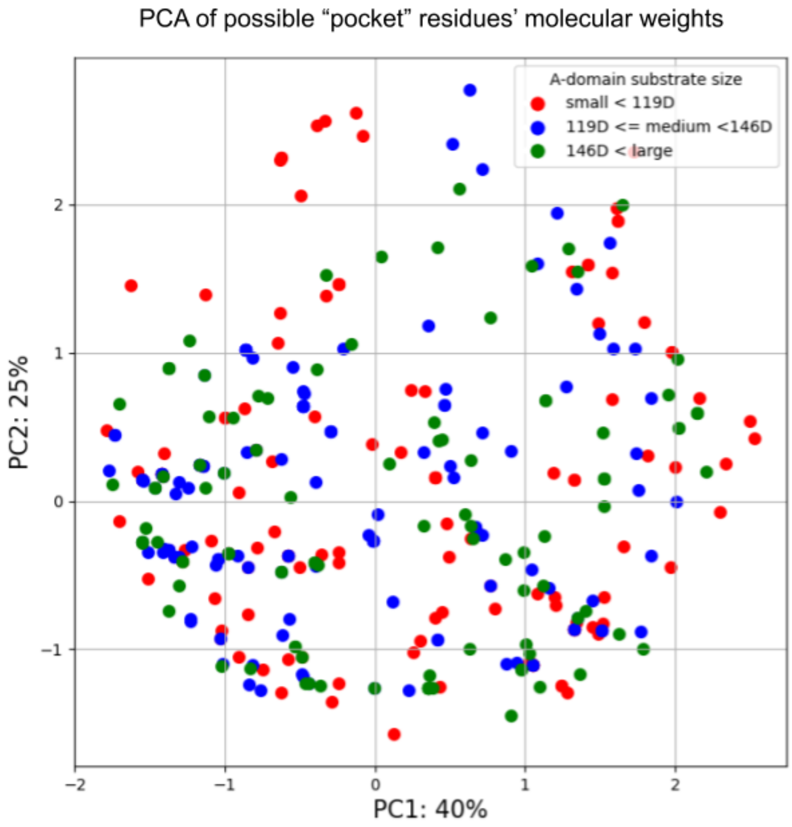

Supplementary Figure 15. C domains do not appear have an "A domain like" side chain selection pocket for their acceptor substrates. Principal component analysis of the molecular weight of residues M2917, S2919, Q2921, P2941 and E2950 (possible "pocket") of the C-A linker regions (401 sequences from the MiBiG database), combined with information on the size of the downstream A domain. Principal Component 1 represents 40% of the variation in the dataset and Principal Component 2 represents 25%. If there were some correlation between the "pocket" residues and the substrate, there should have been clustering of same-colored points in the graph. Their absence further supports the results of the correlation analysis conducted with the sum of weights of the "pocket" residues, which indicates no correlation (Spearman's rho: -0.05).

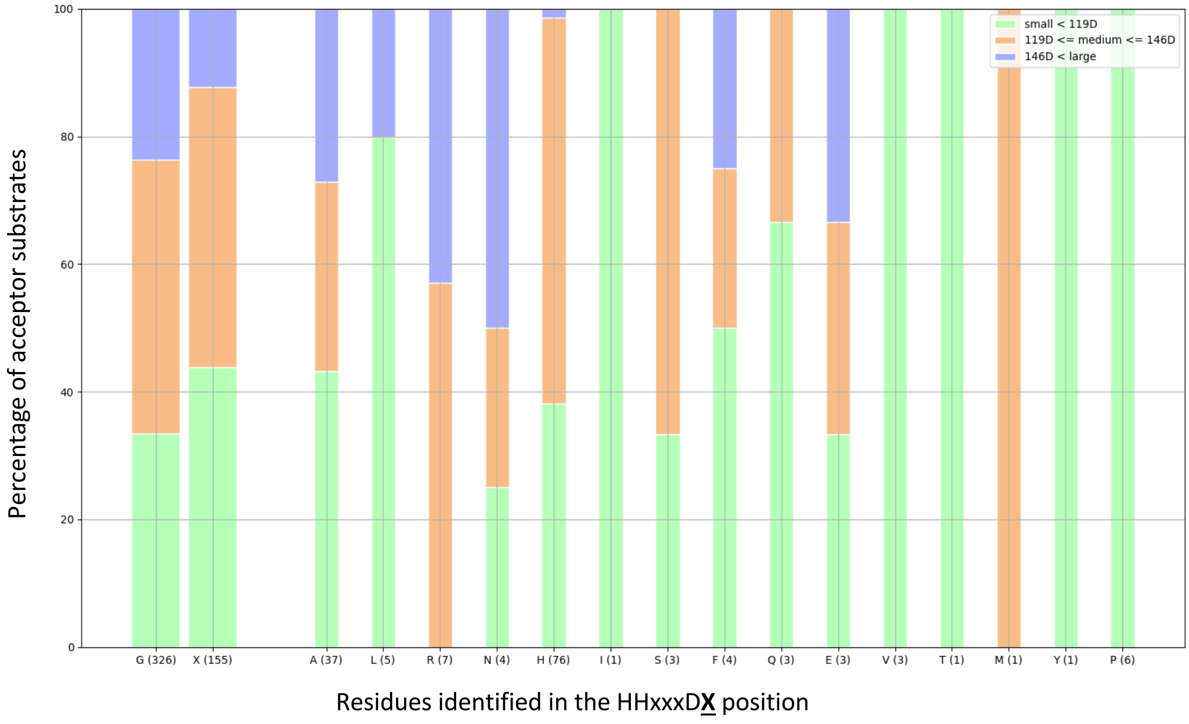

Supplementary Figure 16. Stacked bar plots of substrate size percentage by mutation in the HHxxxDX motif. Left hand thicker bars show the acceptor substrates for C domains containing either the canonical HHxxxDG motif or those for a modified HHxxxDX motif. Each bar indicates the proportion of small (green), medium (orange) and large (blue) acceptor substrates for C domains bearing the specific HHxxxDX motif. The thinner bars represent specific residues found in C domains bearing a modified HHxxxDX motif. The numbers in the parentheses show the quantity of sequences taken into account for each case. There is a higher percentage of small acceptor substrates in the modified motifs in total, as well as in most individual cases.

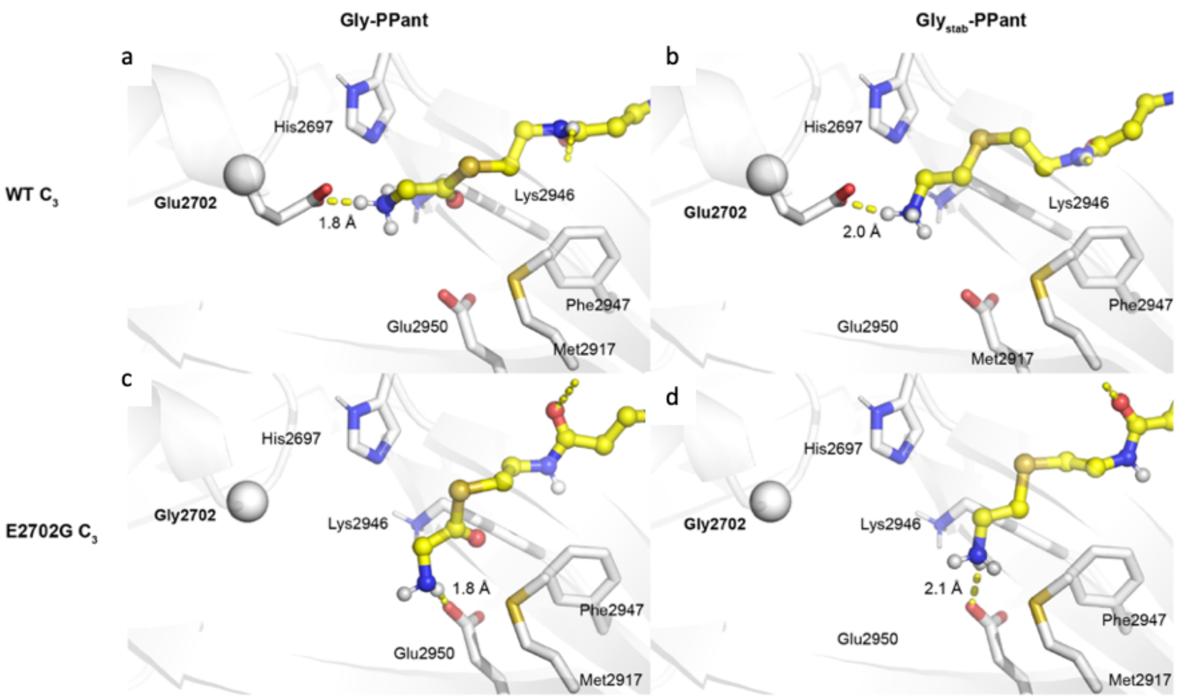

Supplementary Figure 17. Glycine-PPant and Gly$_{stab}$-PPant computationally docked into WT and E2702G C$_3$ domains. Top-scoring computational docking poses of glycine-PPant (left) and Gly$_{stab}$-PPant (right) in WT C$_3$ domain (top, PDB ID 7KW2) and a model of the E2702G mutant C$_3$ domain (bottom). Removal of the key Glu2702 residue (to the more common Gly2702) leads to top-scoring poses in which the terminal amine is no longer positioned near the putative catalytic residue, His2697. Hydrogen bond distances indicated.

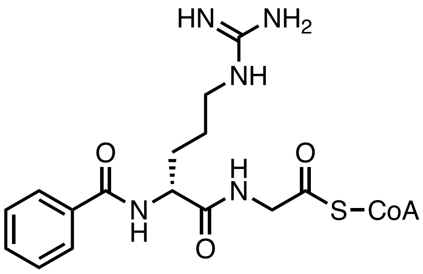

Supplementary Figure 18. Synthesis and characterization of BA-D-Arg-Gly-CoA. Synthesized according to the peptidyl-CoA synthesis protocol. ^{1}H NMR (600 MHz, D_2O): δ 8.65 (s, 1H), 8.41 (s, 1H), 7.78 – 7.74 (m, 2H), 7.61 – 7.57 (m, 1H), 7.50 – 7.47 (m, 2H), 6.20 (d, *J* = 5.5 Hz, 1H), 4.92 – 4.88 (m, 2H), 4.61 (s, 1H), 4.59 – 4.56 (m, 1H), 4.34 – 4.26 (m, 2H), 4.22 – 4.14 (m, 2H), 3.90 – 3.88 (m, 1H), 3.66 – 3.63 (m, 1H), 3.43 (t, *J* = 6.5 Hz, 2H), 3.34 (t, *J* = 6.0 Hz, 2H), 3.27 (t, *J* = 7.0 Hz, 2H), 3.04 (t, *J* = 6.5 Hz, 2H), 2.40 (t, *J* = 6.5 Hz, 2H), 2.07 – 2.01 (m, 1H), 1.98 -1.90 (m, 1H), 1.83 – 1.72 (m, 2H), 0.96 (s, 3H), 0.84 (s, 3H); HRMS (ESI): calculated for $C_{36}H_{56}N_{12}O_{19}P_3S^{2+}$ $[M+H]^{2+}$: 543.1393, found: 543.1399 (Δ 0.3 ppm).

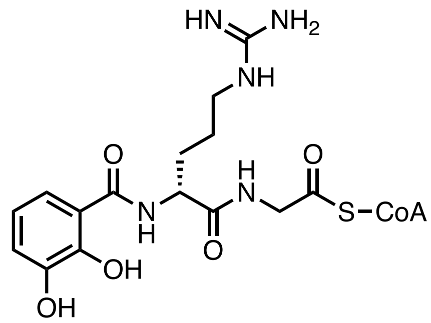

Supplementary Figure 19. Synthesis and characterization of DHB-D-Arg-Gly-CoA. Synthesized according to the peptidyl-CoA synthesis protocol. ^{1}H NMR (400 MHz, D_2O): δ 8.37 (s, 1H), 8.05 (s, 1H), 7.12 – 7.08 (m, 1H), 6.90 – 6.85 (m, 1H), 6.68 – 6.64 (s, 1H), 6.02 – 5.98 (m, 1H), 4.79 – 4.75 (m, 1H), 4. 66 – 4.64 (m, 1H), 4.49 - 4.43 (m, 2H), 4.15 – 4.08 (m, 3H), 3.90 (s, 1H), 3.29 – 3.24 (m, 1H), 3.15 – 3.13 (m, 1H), 2.96 -2.93 (s, 2H), 2.27 -2.25 (m, 1H), 1.96 (s, 1H), 0.78 (s, 3H), 0.64 (s, 3H); HRMS (ESI): calculated for $C_{36}H_{56}N_{12}O_{21}P_3S^+$ $[M+H]^+$: 1117.2611, found: 1117.26111 (Δ 0.1 ppm).

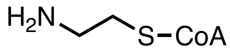

Supplementary Figure 20. Synthesis and characterization of Gly$_{stab}$-CoA. Synthesized according to the modified aminoacyl-CoA synthesis protocol. ^{1}H NMR (600 MHz, D_2O,): δ 8.66 (s, 1H), 8.43 -8.40 (m, 1H), 6.25 (d, *J* = 6.0 Hz, 1H), 4.93 – 4.86 (m, 2H), 4.66 – 4.63 (m, 1H), 4.34 – 4.25 (m, 2H), 4.08 – 4.05 (m, 1H), 3.93 – 3.91 (m, 1H), 3.90 – 3.86 (m ,1H), 3.65 – 3.61 (m, 1H), 3.55 – 3.51 (m, 3H), 3.50 – 3.45 (m, 1H), 3.42 – 3.39 (m, 1H), 3.38 – 3.35 (m, 1H), 3.28 – 3.24 (m, 1H), 2.92 – 2.88 (m, 1H), 2.87 -2.81 (m, 1H), 2.54 – 2.50 (m, 2H), 0.97 - 0.94 (m, 3H), 0.85 – 0.82 (m, 3H); HRMS (ESI): calculated for $C_{23}H_{42}N_8O_{16}P_3S^+$ $[M+H]^+$: 811.1647, found: 811.1650 (Δ 0.3 ppm).

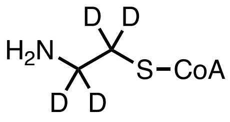

Supplementary Figure 21. Synthesis and characterization of *d*$_4$-Gly$_{stab}$-CoA. Synthesized according to the modified aminoacyl-CoA synthesis protocol. ^{1}H NMR (600 MHz, D_2O): δ 8.58 – 8.55 (m, 1H), 8.48 – 8.44 (m, 1H), 8.30 – 8.27 (m, 1H), 6.20 – 6.19 (m, 1H), 4.88 – 4.84 (m, 2H), 4.59 (s, 2H), 4.25 (s, 2H), 4.05 – 4.02 (m, 1H), 3.85 – 3.80 (m, 2H), 3.59 – 3.55 (m, 2H), 3.39 – 3.34 (m, 3H), 2.69 (t, *J* = 7.0 Hz, 2H), 2.47 (t, *J* = 6.5 Hz, 2H), 0.90 (s, 3H), 0.79 (s, 3H); HRMS (ESI): calculated for $C_{23}H_{37}D_4N_8O_{16}P_3S^+$ $[M+H]^+$: 815.1898, found: 815.1896 (Δ 0.2 ppm).

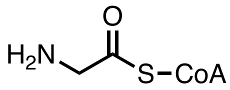

Supplementary Figure 22. Synthesis and characterization of Gly-CoA. Synthesized according to the aminoacyl-CoA synthesis protocol. ^{1}H NMR (600 MHz, D_2O): δ 8.70 (s, 1H), 8.47 (s, 1H), 6.28 – 5.24 (m, 1H), 4.93 – 4.88 (m, 2H), 4.63 (s, 1H), 4.3 – 4.2 (m, 2H), 4.19 (s, 2H), 4.06 (m, 1H), 3.88 (dd, *J* = 9.5, 4.5 Hz, 1H), 3.65 (dd, *J* = 9.5, 4.5 Hz, 1H), 3.54 – 3.47 (m, 2H), 3.44 (t, *J* = 6.5 Hz, 2H), 3.19 (t, *J* = 6.0 Hz, 2H), 2.48 (t, *J* = 6.5 Hz, 2H), 0.97 (s, 3H), 0.87 (s, 3H); HRMS (ESI): calculated for $C_{23}H_{40}N_8O_{17}P_3S^+$ $[M+H]^+$: 825.1439, found: 825.14537 (Δ 1.7 ppm).

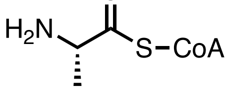

Supplementary Figure 23. Synthesis and characterization of Ala-CoA. Synthesized according to the aminoacyl-CoA synthesis protocol. ^{1}H NMR (600 MHz, D_2O): δ 8.70 (s, 1H), 8.65 (s, 1H), 8.47 (s, 1H), 6.27 – 6.23 (m, 1H),

5.19 – 5.14 (m, 1H), 4.94 – 4.89 (m, 2H), 4.65 – 4.62 (m, 1H), 4.39 (q, J = 7.0 Hz, 1H), 4.07 – 4.05 (m, 1H), 3.90 – 3.86 (m, 1H), 3.67 – 3.61 (m, 1H), 3.55 – 3.46 (m, 2H), 3.43 (t, J = 6.5 Hz, 2H), 3.24 – 3.12 (m, 2H), 2.48 (t, J = 6.5 Hz, 2H), 1.62 (d, J = 7.0 Hz, 3H), 0.97 (s, 3H), 0.87 (s, 3H); HRMS (ESI): calculated for $C_{24}H_{42}N_8O_{17}P_3S^+$ $[M-H]^-$: 837.1450, found: 837.14505 (Δ 0.6 ppm).

L-Phe- and L-Leu-CoA were synthesized as previously reported; spectra were identical to those reported.[19,20]

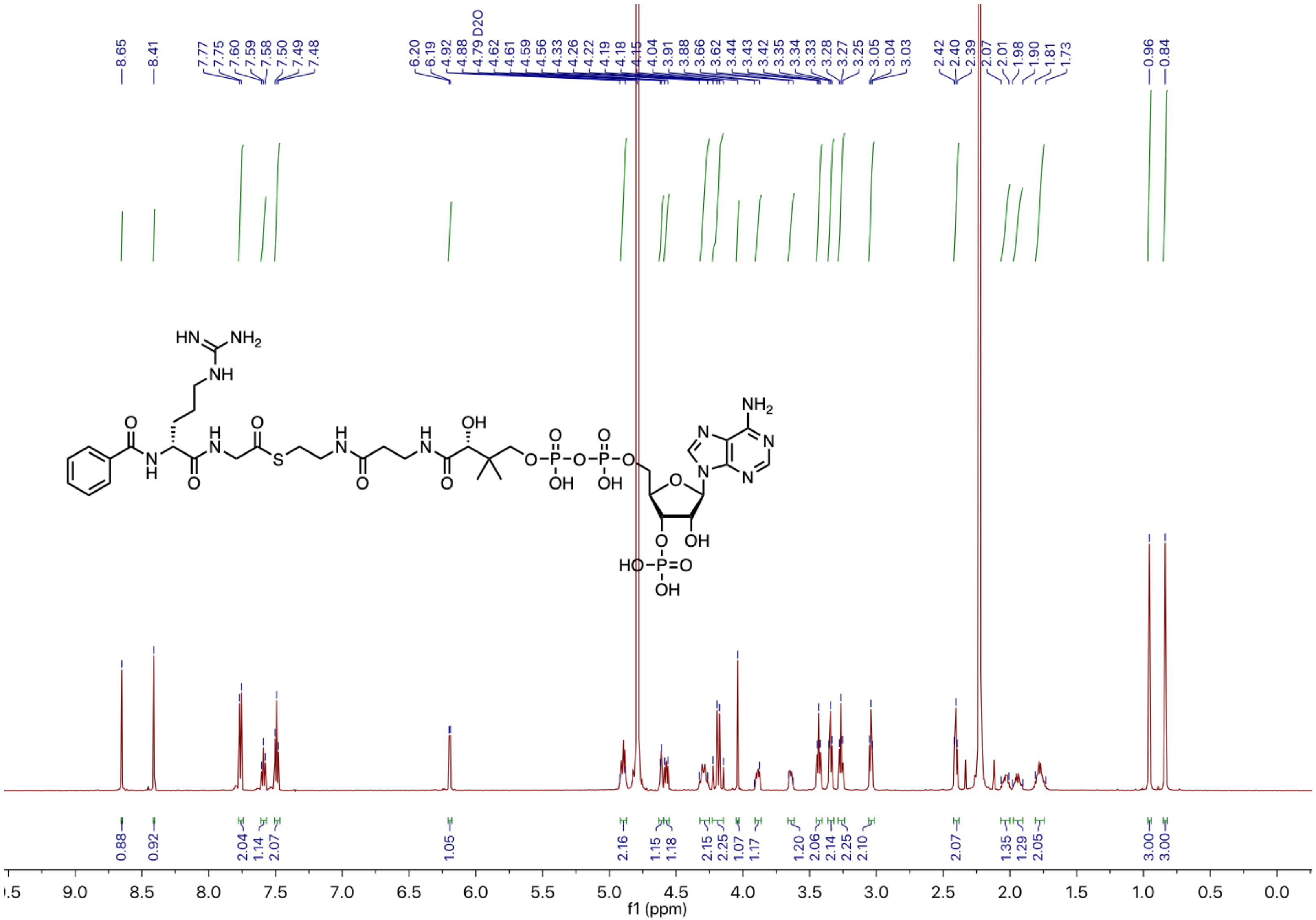

Supplementary Figure 24. ^{1}H NMR spectrum of BA-D-Arg-Gly CoA.

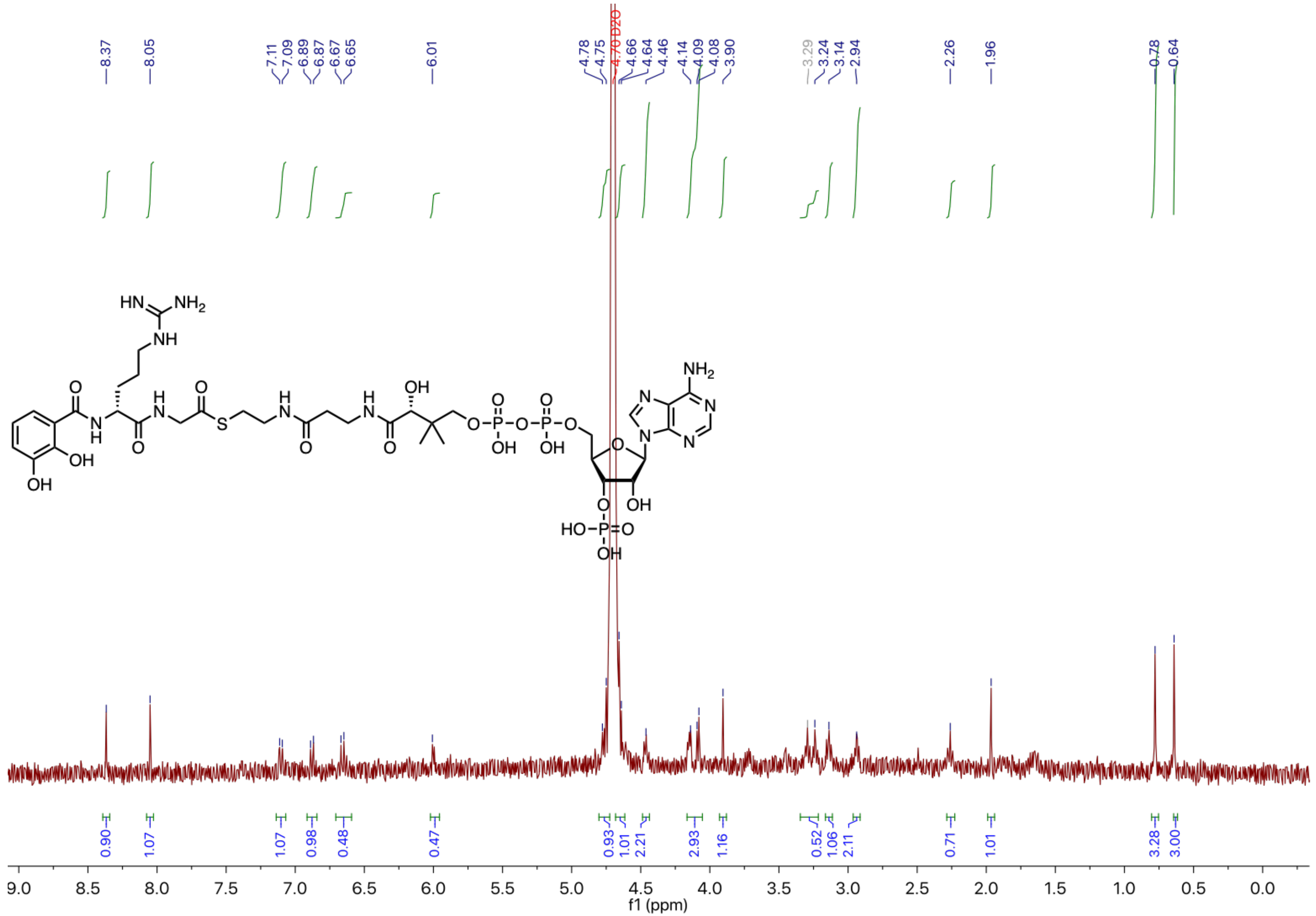

Supplementary Figure 25. ^{1}H NMR spectrum of DHB-D-Arg-Gly CoA.

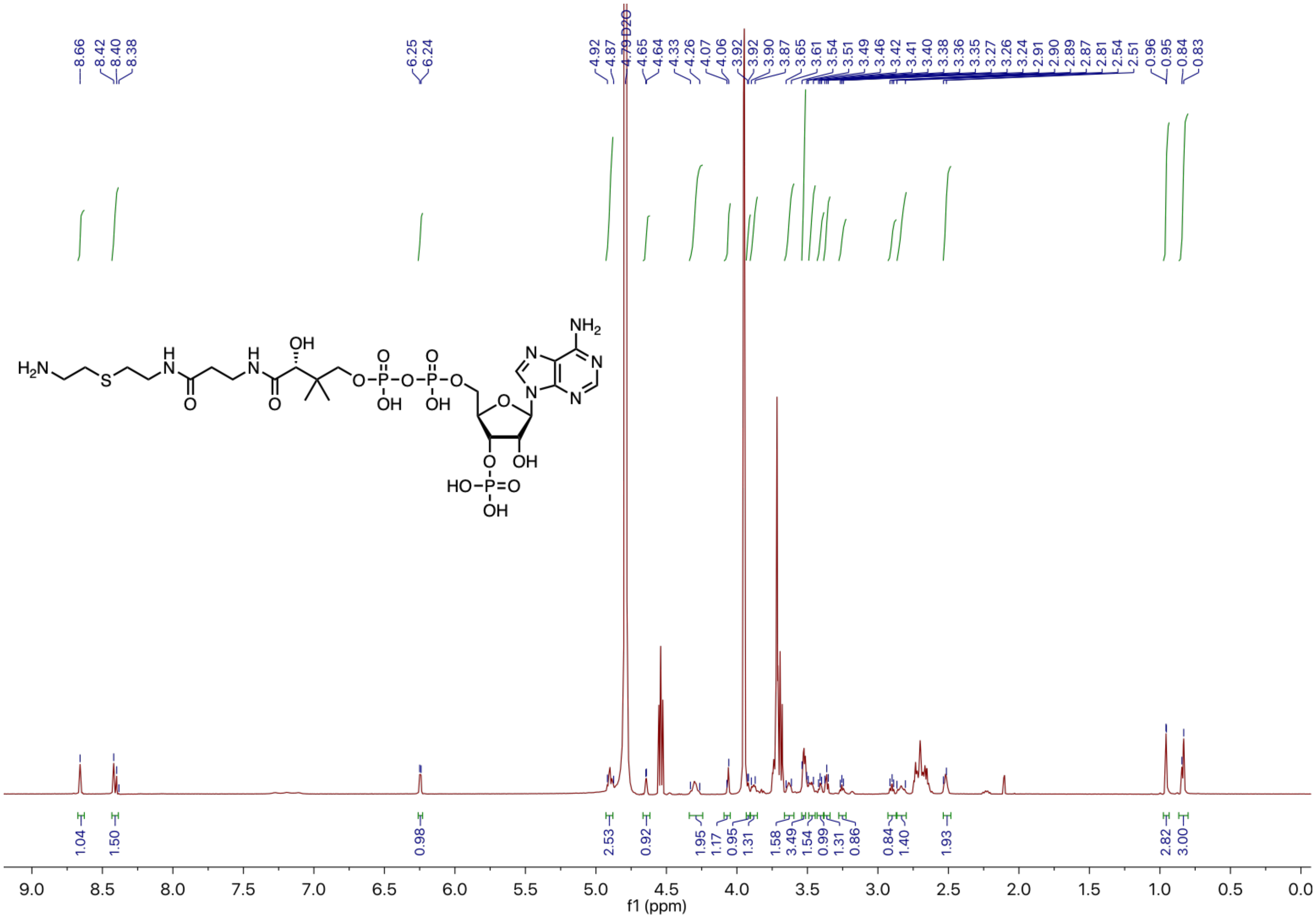

Supplementary Figure 26. ^{1}H NMR spectrum of Gly$_{stab}$-CoA.

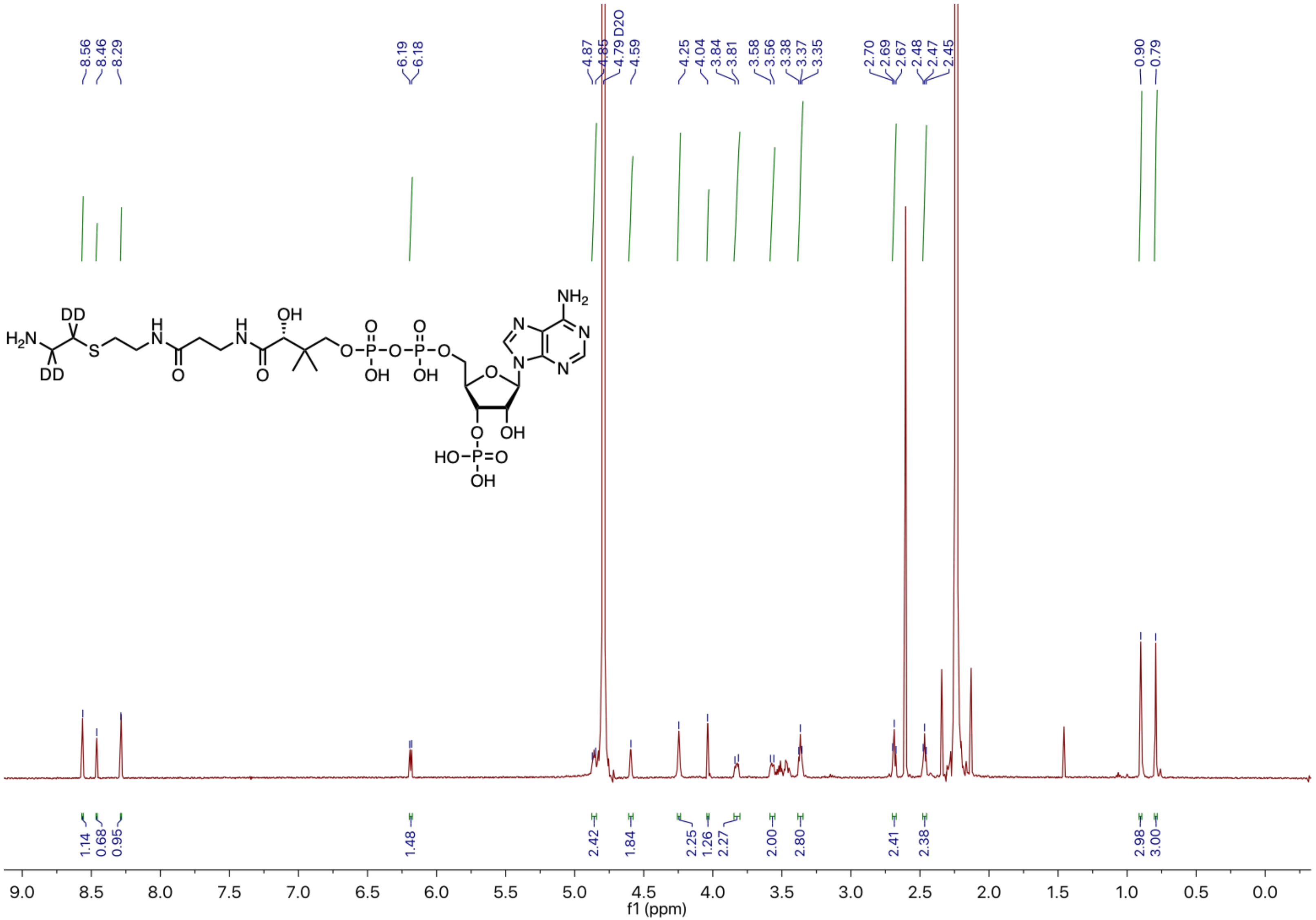

Supplementary Figure 27. **^{1}H NMR spectrum of *d*$_4$-Gly$_{stab}$-CoA.**

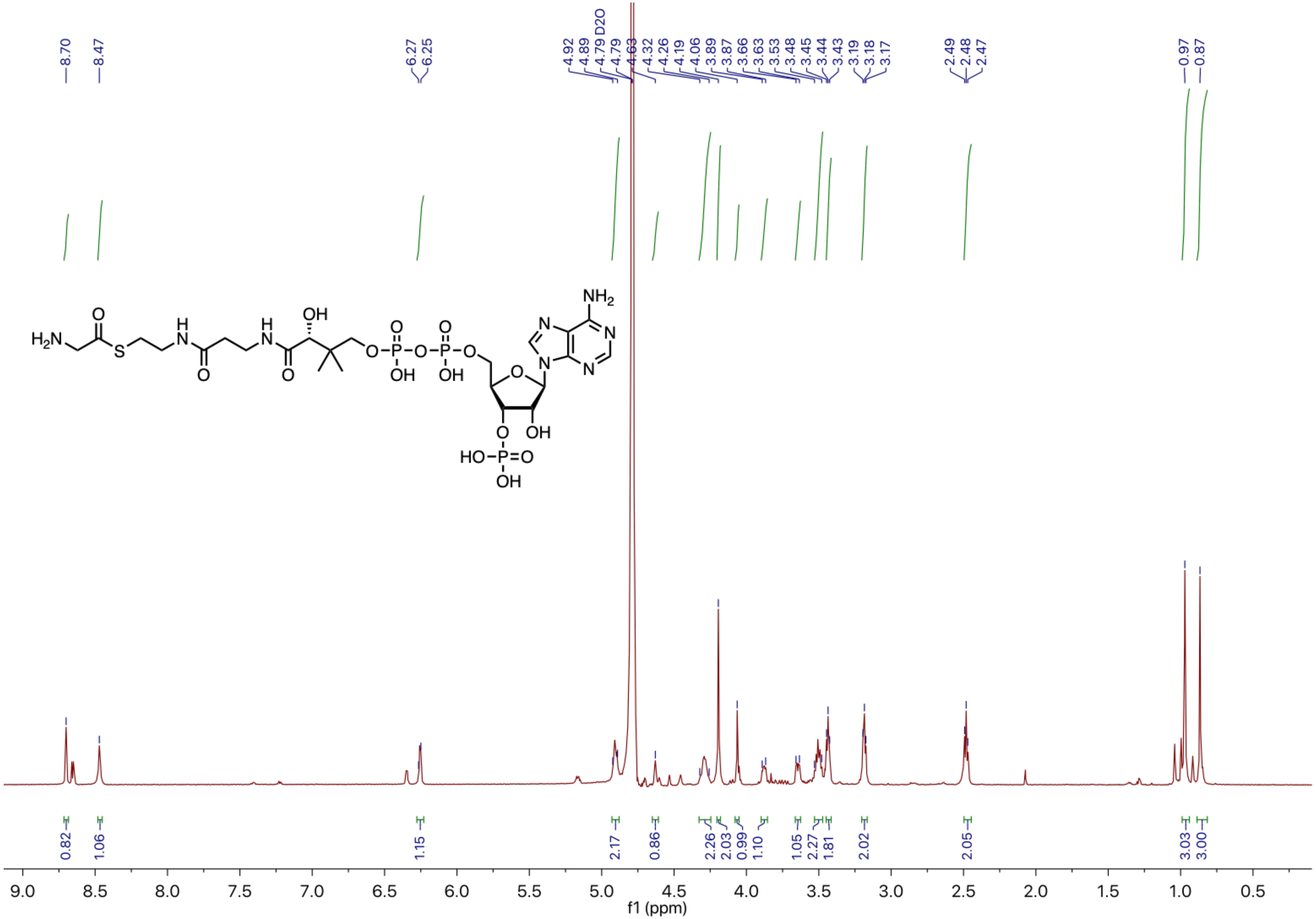

Supplementary Figure 28. ^{1}H NMR spectrum of Gly-CoA.

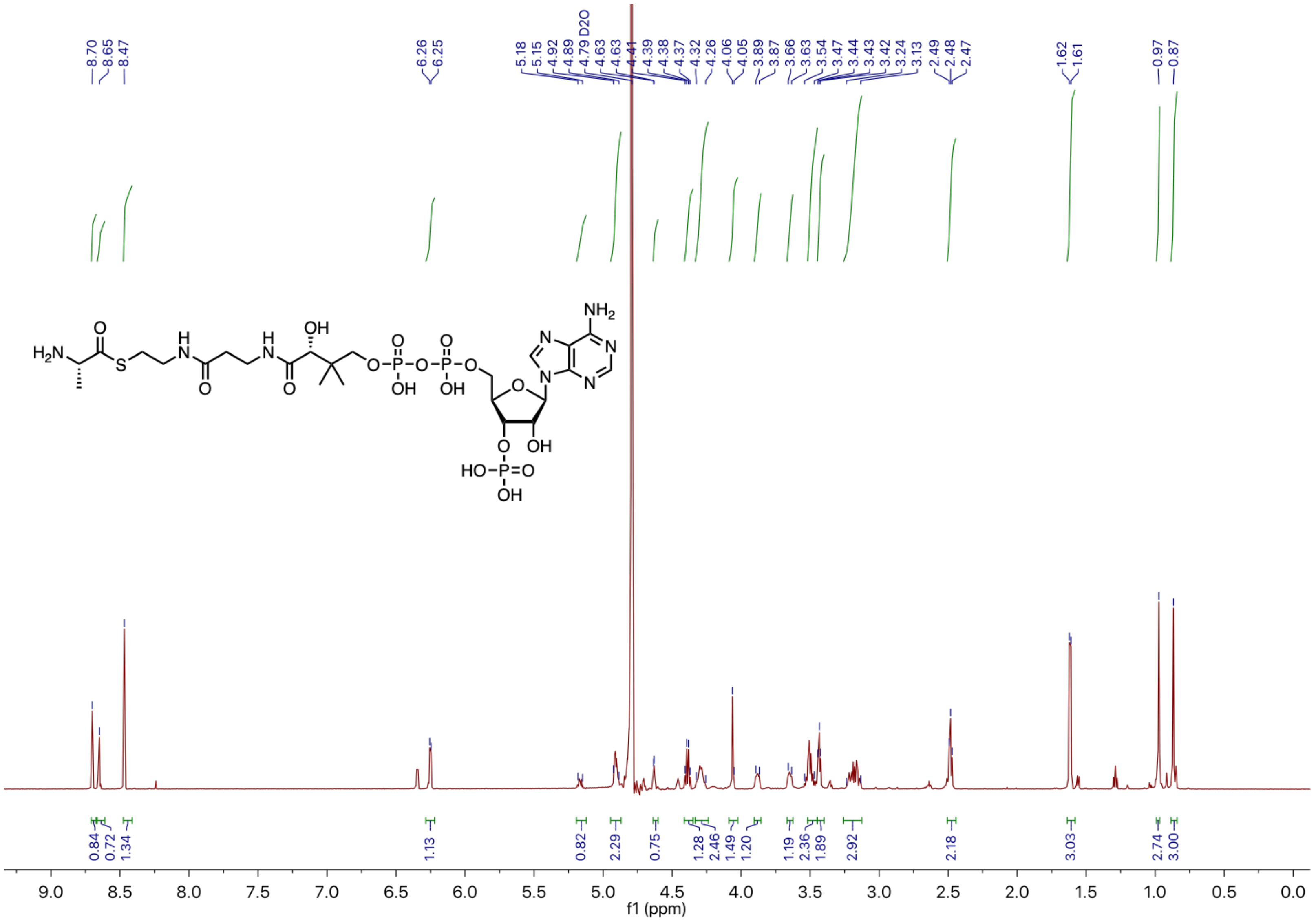

Supplementary Figure 29. **^{1}H NMR spectrum of Ala-CoA.**

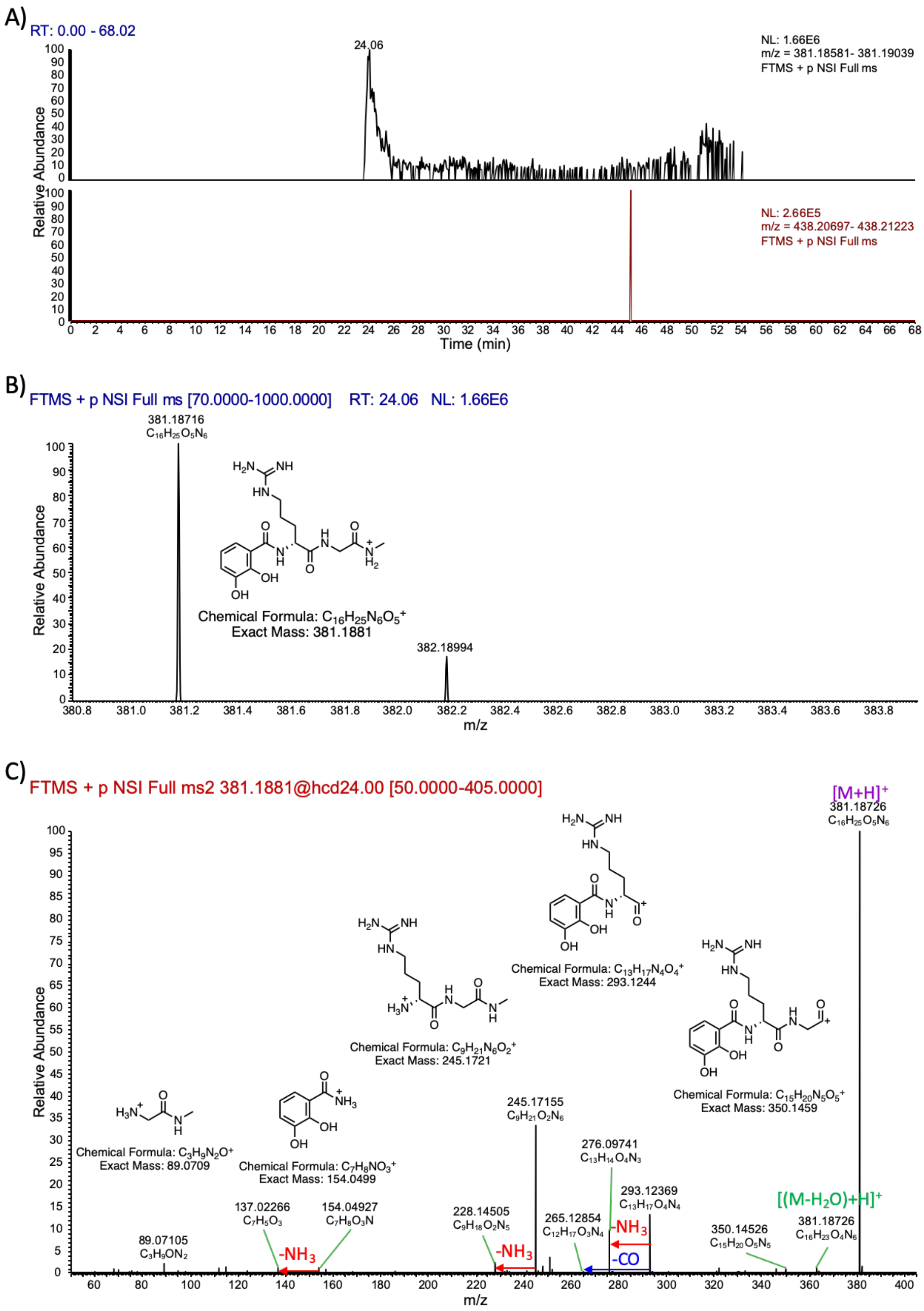

Supplementary Figure 30. LC-HRMS analyses for reconstitution of PCP_2-C_3::PCP_3 WT without SpyCatcher and SpyTag. Experiments utilized DHB-D-Arg-Gly as the donor substrate and Gly as the acceptor substrate. A) Extracted ion chromatograms for masses corresponding to the donor tripeptide (upper) and product tetrapeptide (lower) (Orbitrap Fusion, $[M+H]^+$). B) Accurate mass and isotopic distribution of DHB-D-Arg-Gly donor. C) MS^2 fragmentation for DHB-D-Arg-Gly donor.

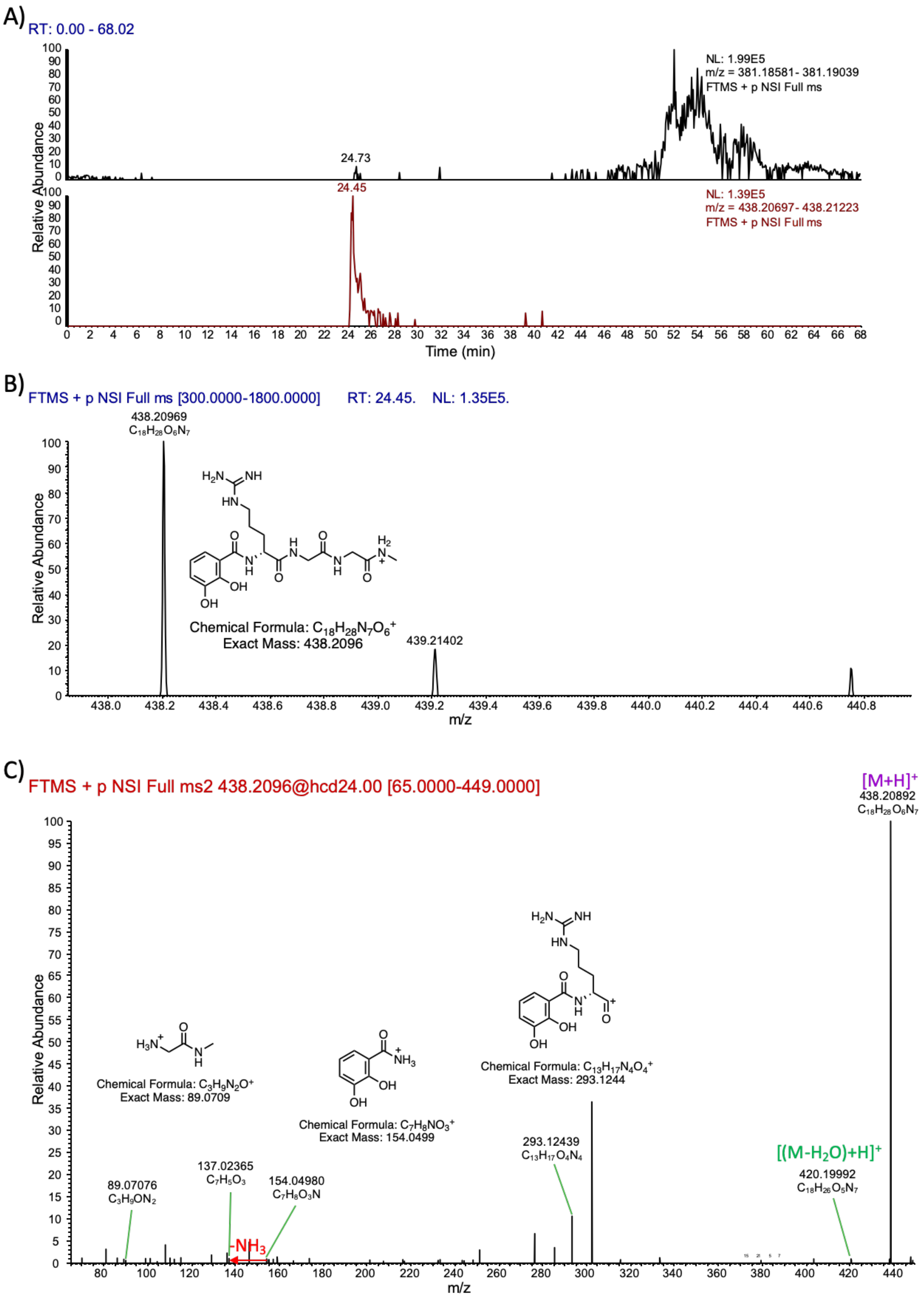

Supplementary Figure 31. LC-HRMS analyses for reconstitution of PCP_2-C_3::PCP_3 WT using SpyCatcher and SpyTag. Experiments utilized DHB-D-Arg-Gly as the donor substrate and Gly as the acceptor substrate. A) Extracted ion chromatograms for masses corresponding to the donor tripeptide (upper) and product tetrapeptide (lower) (Orbitrap Fusion, $[M+H]^+$). B) Accurate mass and isotopic distribution of DHB-D-Arg-Gly-Gly product. C) MS^2 fragmentation for DHB-D-Arg-Gly-Gly product.

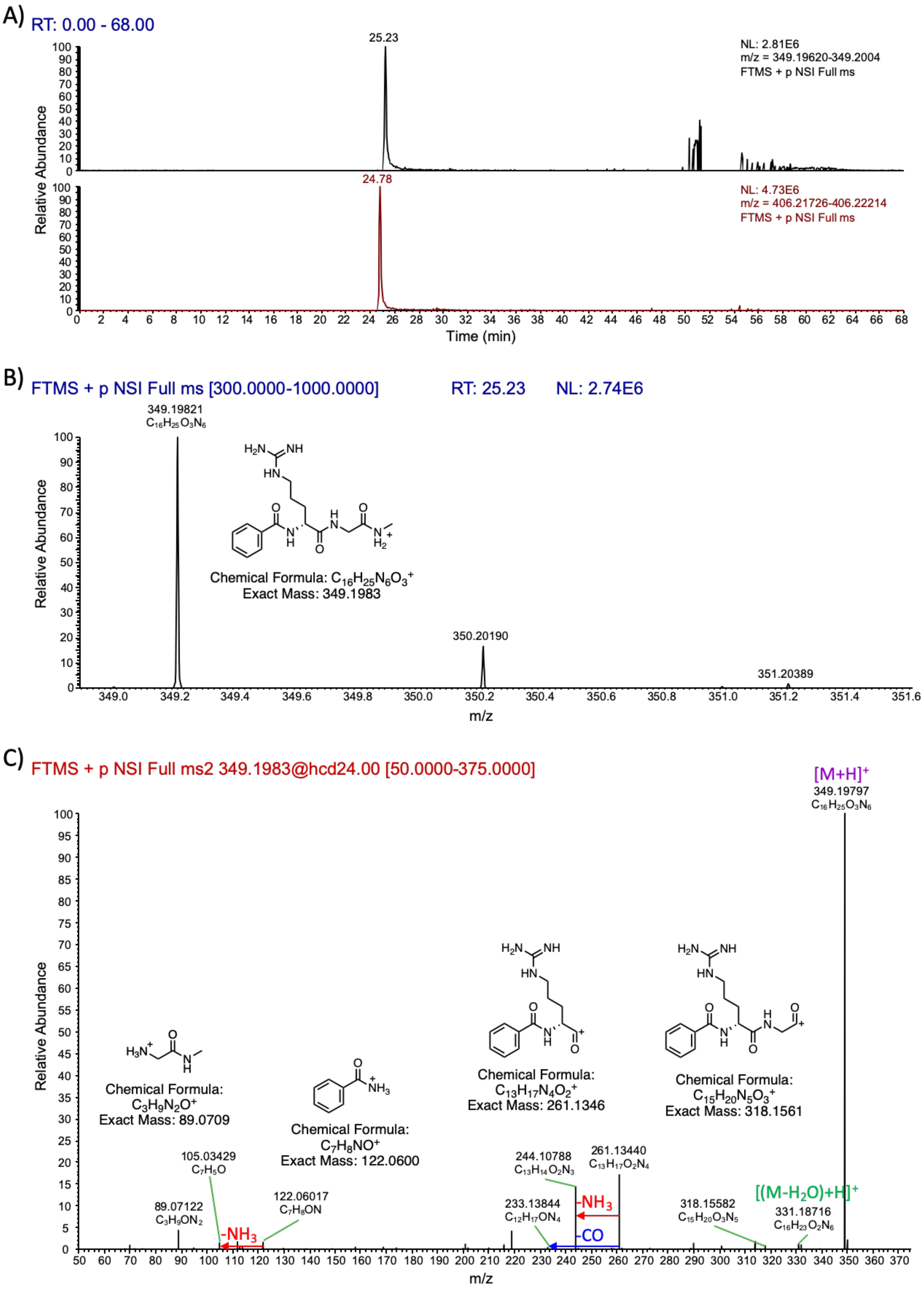
A)
RT: 0.00 - 68.00
25.23
NL: 2.81E6
m/z = 349.19620-349.2004
FTMS + p NSI Full ms
24.78
NL: 4.73E6
m/z = 406.21726-406.22214
FTMS + p NSI Full ms
Relative Abundance
Time (min)
B)
FTMS + p NSI Full ms [300.0000-1000.0000]
RT: 25.23
NL: 2.74E6
349.19821
C16H25O3N6
Chemical Formula: C16H25N6O3+
Exact Mass: 349.1983
350.20190
351.20389
Relative Abundance
m/z
C)
FTMS + p NSI Full ms2 349.1983@hcd24.00 [50.0000-375.0000]
[M+H]+
349.19797
C16H25O3N6
Chemical Formula: C3H9N2O+
Exact Mass: 89.0709
Chemical Formula: C7H8NO+
Exact Mass: 122.0600
Chemical Formula: C13H17N4O2+
Exact Mass: 261.1346
Chemical Formula: C15H20N5O3+
Exact Mass: 318.1561
105.03429
C7H5O
89.07122
C3H9ON2
122.06017
C7H8ON
-NH3
244.10788
C13H14O2N3
261.13440
C13H17O2N4
233.13844
C12H17ON4
-NH3
-CO
318.15582
C15H20O3N5
[(M-H2O)+H]+
331.18716
C16H23O2N6
Relative Abundance
m/z

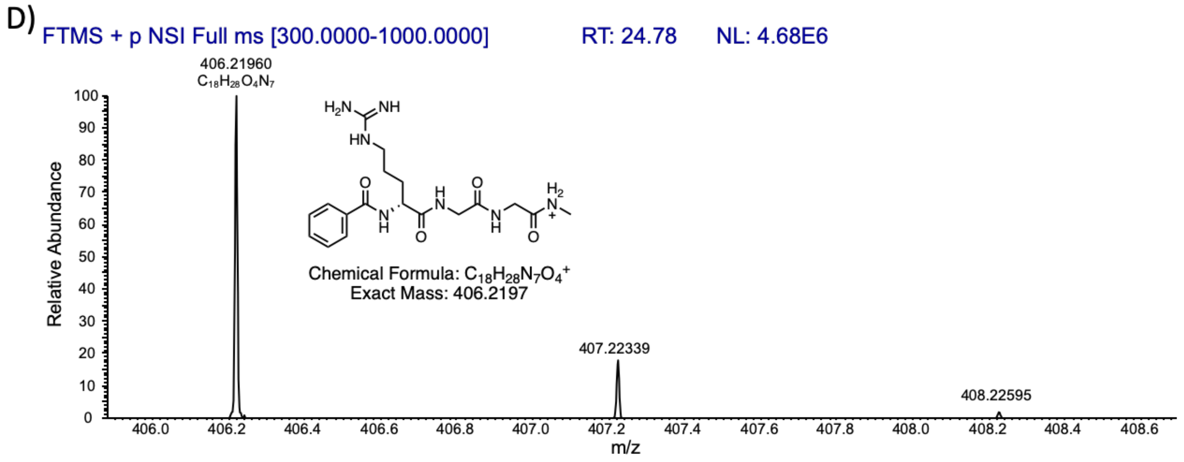

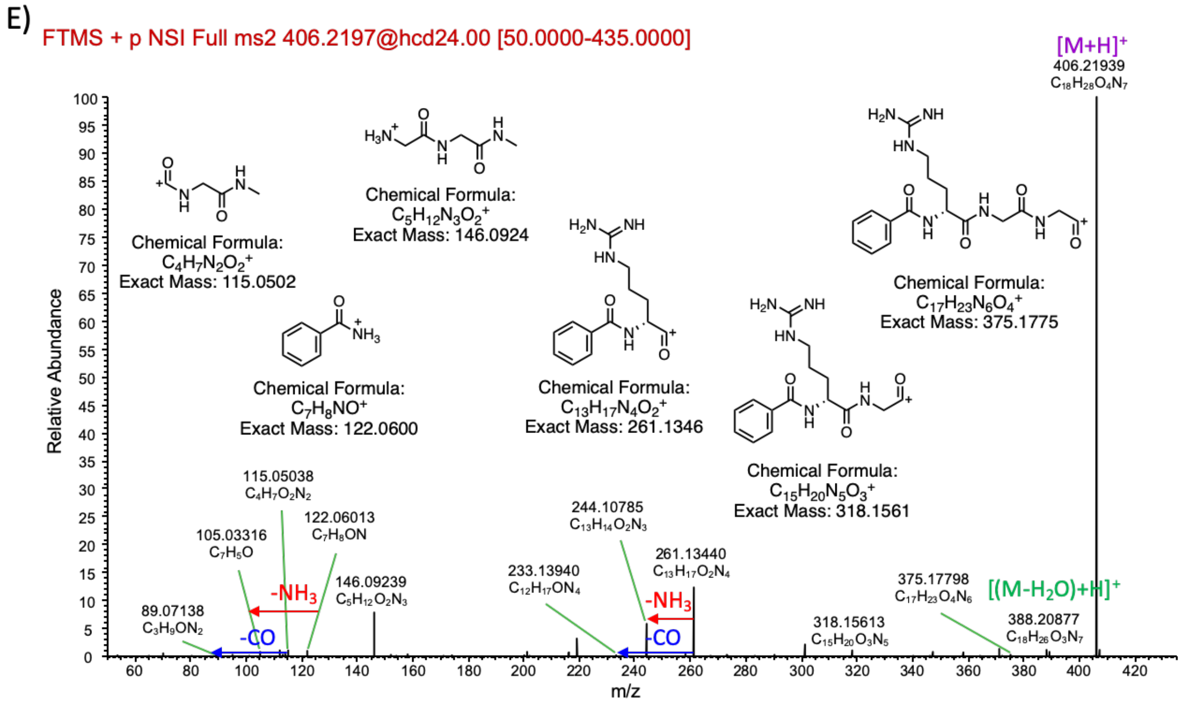

Supplementary Figure 32. LC-HRMS analyses for reconstitution of PCP_2-C_3::PCP_3 WT using SpyCatcher and SpyTag. Experiments utilized BA-D-Arg-Gly as the donor substrate and Gly as the acceptor substrate. A) Extracted ion chromatograms for masses corresponding to the donor tripeptide (upper) and product tetrapeptide (lower) (Orbitrap Fusion, $[M+H]^+$). B) Accurate mass and isotopic distribution of BA-D-Arg-Gly donor. C) MS^2 fragmentation for BA-D-Arg-Gly donor. D) Accurate mass and isotopic distribution of BA-D-Arg-Gly-Gly product. E) MS^2 fragmentation for BA-D-Arg-Gly-Gly product.

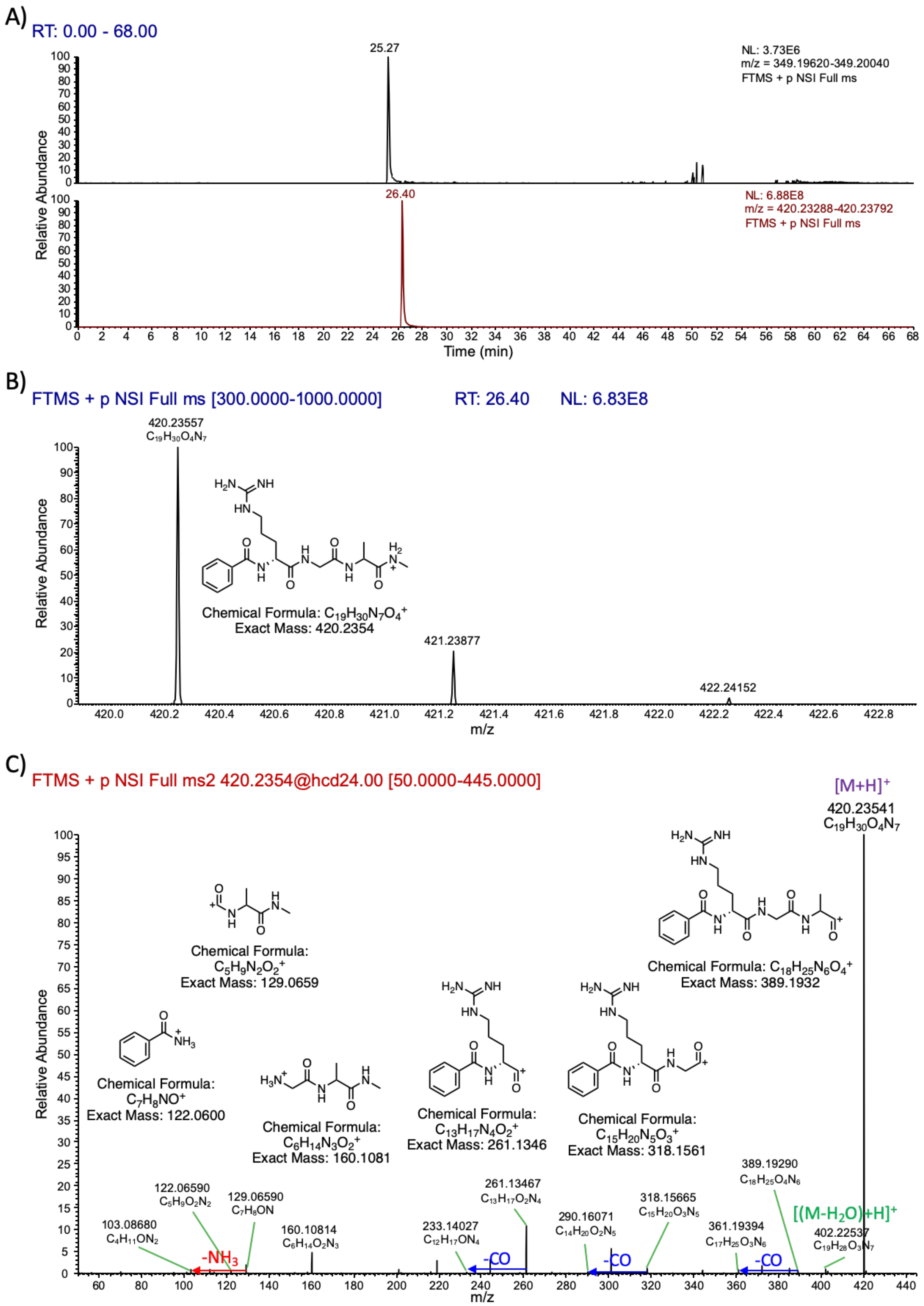

Supplementary Figure 33. LC-HRMS analyses for reconstitution of PCP$_2$-C$_3$::PCP$_3$ WT using SpyCatcher and SpyTag. Experiments utilized BA-D-Arg-Gly as the donor substrate and Ala as the acceptor substrate. A) Extracted ion chromatograms for masses corresponding to the donor tripeptide (upper) and product tetrapeptide (lower) (Orbitrap Fusion, $[M+H]^+$). B) Accurate mass and isotopic distribution of BA-D-Arg-Gly-Ala product. C) MS^2 fragmentation for BA-D-Arg-Gly-Ala product.

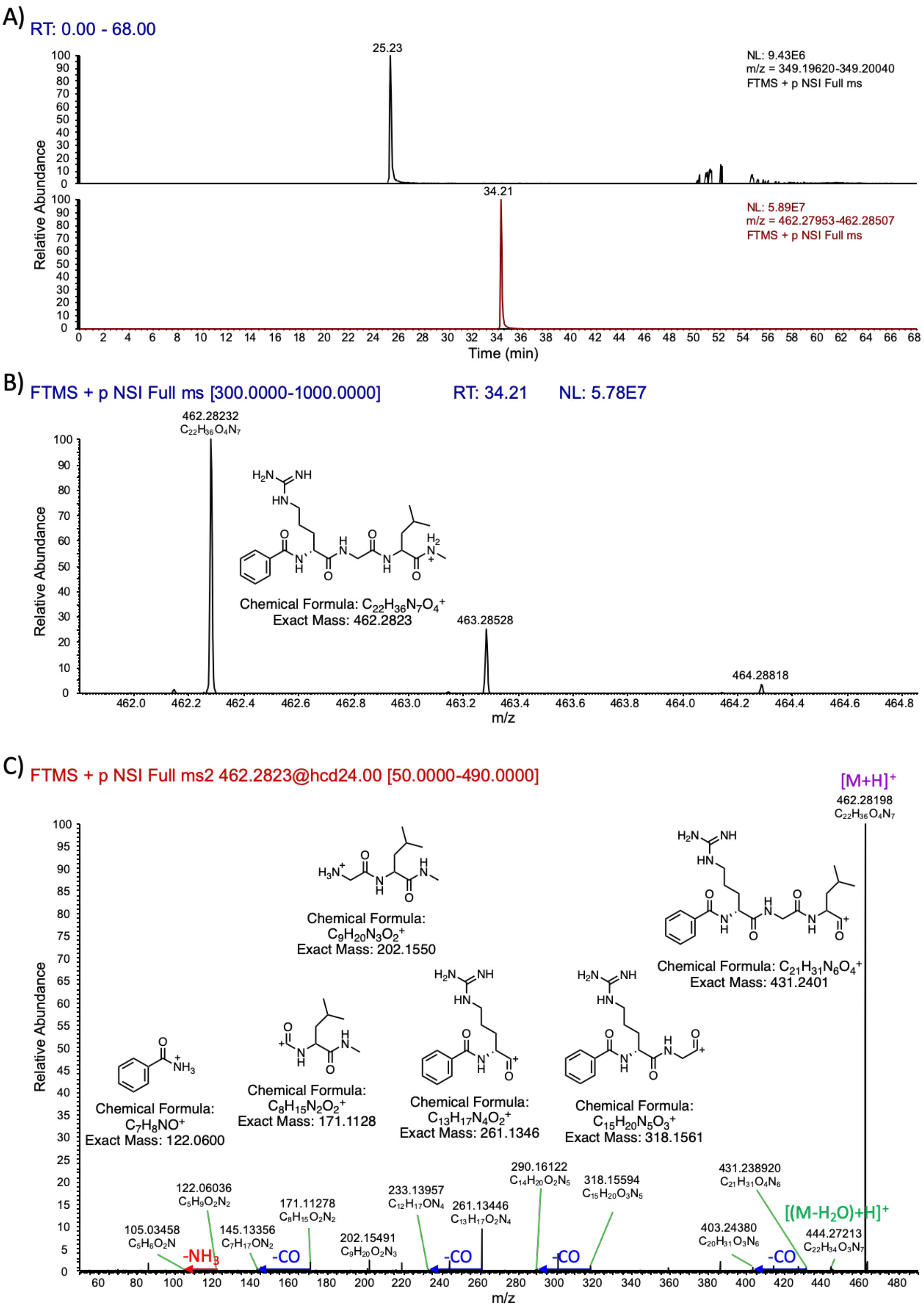

Supplementary Figure 34. LC-HRMS analyses for reconstitution of PCP_2-C_3::PCP_3 WT using SpyCatcher and SpyTag. Experiments utilized BA-D-Arg-Gly as the donor substrate and Leu as the acceptor substrate. A) Extracted ion chromatograms for masses corresponding to the donor tripeptide (upper) and product tetrapeptide (lower) (Orbitrap Fusion, $[M+H]^+$). B) Accurate mass and isotopic distribution of BA-D-Arg-Gly-Leu product. C) MS^2 fragmentation for BA-D-Arg-Gly-Leu product.

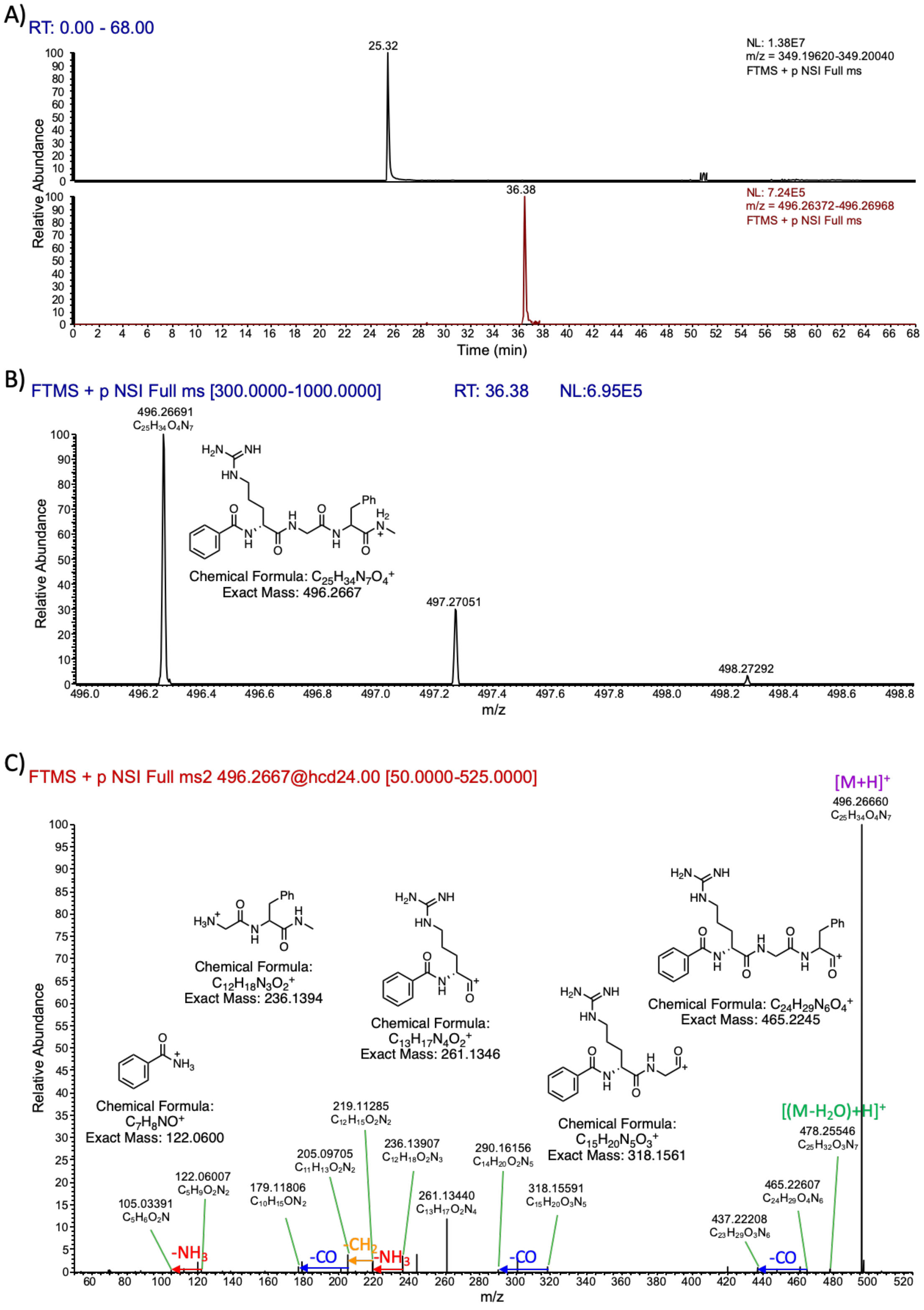

Supplementary Figure 35. LC-HRMS analyses for reconstitution of PCP$_2$-C$_3$::PCP$_3$ WT using SpyCatcher and SpyTag. Experiments utilized BA-D-Arg-Gly as the donor substrate and Phe as the acceptor substrate. A) Extracted ion chromatograms for masses corresponding to the donor tripeptide

(upper) and product tetrapeptide (lower) (Orbitrap Fusion, $[M+H]^+$). B) Accurate mass and isotopic distribution of BA-D-Arg-Gly-Phe product. C) MS^2 fragmentation for BA-D-Arg-Gly-Phe product.

Supplementary References

1 Holm, L. & Rosenström, P. Dali server: conservation mapping in 3D. *Nucleic Acids Res.* **38**, W545-W549, (2010).

2 Drake, E. J. *et al.* Structures of two distinct conformations of holo-non-ribosomal peptide synthetases. *Nature* **529**, 235-238, (2016).

3 Tufar, P. *et al.* Crystal Structure of a PCP/Sfp Complex Reveals the Structural Basis for Carrier Protein Posttranslational Modification. *Chem. Biol.* **21**, 552-562, (2014).

4 Lohman, J. R. *et al.* The crystal structure of BlmI as a model for nonribosomal peptide synthetase peptidyl carrier proteins. *Proteins Struct. Func. Bioinf.* **82**, 1210-1218, (2014).

5 Liu, Y., Zheng, T. & Bruner, Steven D. Structural Basis for Phosphopantetheinyl Carrier Domain Interactions in the Terminal Module of Nonribosomal Peptide Synthetases. *Chem. Biol.* **18**, 1482-1488, (2011).

6 Samel, S. A., Schoenafinger, G., Knappe, T. A., Marahiel, M. A. & Essen, L.-O. Structural and Functional Insights into a Peptide Bond-Forming Bidomain from a Nonribosomal Peptide Synthetase. *Structure* **15**, 781-792, (2007).

7 Haslinger, K., Peschke, M., Brieke, C., Maximowitsch, E. & Cryle, M. J. X-domain of peptide synthetases recruits oxygenases crucial for glycopeptide biosynthesis. *Nature* **521**, 105-109, (2015).

8 Reimer, J. M. *et al.* Structures of a dimodular nonribosomal peptide synthetase reveal conformational flexibility. *Science* **366**, eaaw4388, (2019).

9 Greule, A. *et al.* Kistamicin biosynthesis reveals the biosynthetic requirements for production of highly crosslinked glycopeptide antibiotics. *Nature Commun.* **10**, 2613, (2019).

10 Krissinel, E. & Henrick, K. Inference of Macromolecular Assemblies from Crystalline State. *J. Mol. Biol.* **372**, 774-797, (2007).

11 Frisch, M. J. T., G. W.; Schlegel, H. B.; Scuseria, G. E.; Robb, M. A.; Cheeseman, J. R.; Scalmani, G.; Barone, V.; Mennucci, B.; Petersson, G. A.; Nakatsuji, H.; Caricato, M.; Li, X.; Hratchian, H. P.; Izmaylov, A. F.; Bloino, J.; Zheng, G.; Sonnenberg, J. L.; Hada, M.; Ehara, M.; Toyota, K.; Fukuda, R.; Hasegawa, J.; Ishida, M.; Nakajima, T.; Honda, Y.; Kitao, O.; Nakai, H.; Vreven, T.; Montgomery, J. A., Jr.; Peralta, J. E.; Ogliaro, F.; Bearpark, M.; Heyd, J. J.; Brothers, E.; Kudin, K. N.; Staroverov, V. N.; Kobayashi, R.; Normand, J.; Raghavachari, K.; Rendell, A.; Burant, J. C.; Iyengar, S. S.; Tomasi, J.; Cossi, M.; Rega, N.; Millam, J. M.; Klene, M.; Knox, J. E.; Cross, J. B.; Bakken, V.; Adamo, C.; Jaramillo, J.; Gomperts, R.; Stratmann, R. E.; Yazyev, O.; Austin, A. J.; Cammi, R.; Pomelli, C.; Ochterski, J. W.; Martin, R. L.; Morokuma, K.; Zakrzewski, V. G.; Voth, G. A.; Salvador, P.; Dannenberg, J. J.; Dapprich, S.; Daniels, A. D.; Farkas, Ö.; Foresman, J. B.; Ortiz, J. V.; Cioslowski, J.; Fox, D. J. (Gaussian, Inc., Wallingford, CT, 2013).

12 Lee, C., Yang, W. & Parr, R. G. Development of the Colle-Salvetti correlation-energy formula into a functional of the electron density. *Phys. Rev. B* **37**, 785-789, (1988).

13 Becke, A. D. A new mixing of Hartree–Fock and local density-functional theories. *J. Chem. Phys.* **98**, 1372-1377, (1993).

14 Becke, A. D. Density-functional thermochemistry. III. The role of exact exchange. *J. Chem. Phys.* **98**, 5648-5652, (1993).

15 Stephens, P. J., Devlin, F. J., Chabalowski, C. F. & Frisch, M. J. Ab Initio Calculation of Vibrational Absorption and Circular Dichroism Spectra Using Density Functional Force Fields. *J. Phys. Chem.* **98**, 11623-11627, (1994).

16 Grimme, S., Antony, J., Ehrlich, S. & Krieg, H. A consistent and accurate ab initio parametrization of density functional dispersion correction (DFT-D) for the 94 elements H-Pu. *J. Chem. Phys.* **132**, 154104, (2010).

17 Marenich, A. V., Cramer, C. J. & Truhlar, D. G. Universal Solvation Model Based on Solute Electron Density and on a Continuum Model of the Solvent Defined by the Bulk Dielectric Constant and Atomic Surface Tensions. *J. Phys. Chem. B* **113**, 6378-6396, (2009).

18 Chovancova, E. *et al.* CAVER 3.0: A Tool for the Analysis of Transport Pathways in Dynamic Protein Structures. *PLOS Comp. Biol.* **8**, e1002708, (2012).

19 Uhlmann, S., Süssmuth, R. D. & Cryle, M. J. Cytochrome P450sky Interacts Directly with the Nonribosomal Peptide Synthetase to Generate Three Amino Acid Precursors in Skyllamycin Biosynthesis. *ACS Chem. Biol.* **8**, 2586-2596, (2013).

20 Cryle, M. J., Meinhart, A. & Schlichting, I. Structural Characterization of OxyD, a Cytochrome P450 Involved in b-Hydroxytyrosine Formation in Vancomycin Biosynthesis. *J. Biol. Chem.* **285**, 24562-24574, (2010).
