# Supplementary material for: Structures of a non-ribosomal peptide synthetase condensation domain suggest the basis of substrate selectivity
Source: Nat Commun. 2021 May 4;12:2511. doi: 10.1038/s41467-021-22623-0 (PMC8097023; doi:10.1038/s41467-021-22623-0)
Supplement: Supplementary file 3 — Reporting Summary [file 41467_2021_22623_MOESM3_ESM.pdf]

nature research

Corresponding author(s): Izore and Cryle

Last updated by author(s): Mar 23, 2021

Reporting Summary

Nature Research wishes to improve the reproducibility of the work that we publish. This form provides structure for consistency and transparency in reporting. For further information on Nature Research policies, see our Editorial Policies and the Editorial Policy Checklist.

Statistics

For all statistical analyses, confirm that the following items are present in the figure legend, table legend, main text, or Methods section.

n/a	Confirmed	
☐	☒	The exact sample size (n) for each experimental group/condition, given as a discrete number and unit of measurement
☐	☒	A statement on whether measurements were taken from distinct samples or whether the same sample was measured repeatedly
☒	☐	The statistical test(s) used AND whether they are one- or two-sided<br>*Only common tests should be described solely by name; describe more complex techniques in the Methods section.*
☒	☐	A description of all covariates tested
☐	☒	A description of any assumptions or corrections, such as tests of normality and adjustment for multiple comparisons
☒	☐	A full description of the statistical parameters including central tendency (e.g. means) or other basic estimates (e.g. regression coefficient) AND variation (e.g. standard deviation) or associated estimates of uncertainty (e.g. confidence intervals)
☒	☐	For null hypothesis testing, the test statistic (e.g. F, t, r) with confidence intervals, effect sizes, degrees of freedom and P value noted<br>*Give P values as exact values whenever suitable.*
☒	☐	For Bayesian analysis, information on the choice of priors and Markov chain Monte Carlo settings
☒	☐	For hierarchical and complex designs, identification of the appropriate level for tests and full reporting of outcomes
☒	☐	Estimates of effect sizes (e.g. Cohen's d, Pearson's r), indicating how they were calculated

Our web collection on statistics for biologists contains articles on many of the points above.

Software and code

Policy information about availability of computer code

Data collection	MacroModel 11.7, Gaussian 17, Bruker Avance BACS-400 and BACS600, Data explorer v3,4(Bruker), Lab Solutions (Shimadzu), Unicorn 7 (GE Healthcare),Orbitrap Fusion mass spectrometer (Thermo Scientific). Macromolecular crystallography (MX) beamlines (Australia Synchrotron), MiBIG v2.0
Data analysis	Microsoft Excel v16.20, Pymol v2.2.0, Coot v0.8.9.1, Muscle 3.8.31, WebLogo v 2.8.2 or3.7), SciPy python library v1.4.1, Scikit-learn python library v0.22.2.post1,Matplotlib python library v3.2.1, NumPy python library v1.18.1, Python v3.7.6, Schrödinger Release 2019-1 (including PIPER docking algorithm), Caver 3.0, PISA, Dali, CCP4 v7.0, Phenix 1.18.2-3874, Protein Prospector v5.22.1, QualBrowser (XCalibur 3.0.63, Thermo Scientific), MestReNova v10.0.2, ChemDraw v18.1

For manuscripts utilizing custom algorithms or software that are central to the research but not yet described in published literature, software must be made available to editors and reviewers. We strongly encourage code deposition in a community repository (e.g. GitHub). See the Nature Research guidelines for submitting code & software for further information.

Data

Policy information about availability of data

All manuscripts must include a data availability statement. This statement should provide the following information, where applicable:

- Accession codes, unique identifiers, or web links for publicly available datasets
- A list of figures that have associated raw data
- A description of any restrictions on data availability

Compound characterization by 1H NMR shown in Supplementary Figures 24-29; PPant ejection data shown in Supplementary Figures 13; Representative HRMS traces and MS2 analyses for all turnovers shown in Supplementary Figures 30-35; Crystal structures have been deposited to the protein databank (PDB) under the accession numbers 7KVW [http://doi.org/10.2210/pdb7KVW/pdb], 7KW0 [http://doi.org/10.2210/pdb7KW0/pdb], 7KW2 [http://doi.org/10.2210/pdb7KW2/pdb]

and 7KW3 [http://doi.org/10.2210/pdb7KW3/pdb]. HRMS and PPant ejection data have been deposited to the ProteomeXchange Consortium via the PRIDE partner repository with the dataset identifier PXD024004 [http://proteomecentral.proteomexchange.org/cgi/GetDataset?ID=PXD024004]. All sequences used for statistics and correlation analyses were isolated from the MiBiG database [https://mibig.secondarymetabolites.org/]. Source data for Figure 5d, Supplementary Figure 5c and Supplementary Figure 5f are provided with this paper.

Field-specific reporting

Please select the one below that is the best fit for your research. If you are not sure, read the appropriate sections before making your selection.

☒ Life sciences ☐ Behavioural & social sciences ☐ Ecological, evolutionary & environmental sciences

For a reference copy of the document with all sections, see nature.com/documents/nr-reporting-summary-flat.pdf

Life sciences study design

All studies must disclose on these points even when the disclosure is negative.

Sample size	All enzyme activity assays were performed with the sample of n=3 unless noted
Data exclusions	No data were excluded
Replication	Enzyme assays were all performed in triplicates in a single experiment
Randomization	Data presented in the manuscript are biochemical assays of enzyme function, requiring a rational approach to data collection and analysis; randomization is not appropriate for experimental setup
Blinding	Blinding is not applicable to the biochemical assays performed in this work due to the need for rational design; controls were included

Reporting for specific materials, systems and methods

We require information from authors about some types of materials, experimental systems and methods used in many studies. Here, indicate whether each material, system or method listed is relevant to your study. If you are not sure if a list item applies to your research, read the appropriate section before selecting a response.

Materials & experimental systems

n/a	Involved in the study
☒	☐ Antibodies
☒	☐ Eukaryotic cell lines
☒	☐ Palaeontology and archaeology
☒	☐ Animals and other organisms
☒	☐ Human research participants
☒	☐ Clinical data
☒	☐ Dual use research of concern

Methods

n/a	Involved in the study
☒	☐ ChIP-seq
☒	☐ Flow cytometry
☒	☐ MRI-based neuroimaging
